# Developmental stuttering with common and complex phenotypes

**DOI:** 10.1111/dmcn.70208

**Published:** 2026-02-23

**Authors:** Sarah E. Horton, Daisy A. Shepherd, Stephanie Siemers, Miya St John, Juliette Catherall, Ingrid E. Scheffer, Angela T. Morgan

**Affiliations:** ^1^ Murdoch Children's Research Institute Parkville Victoria Australia; ^2^ Department of Audiology and Speech Pathology University of Melbourne Parkville Victoria Australia; ^3^ Department of Paediatrics University of Melbourne Parkville Victoria Australia; ^4^ Royal Children's Hospital Melbourne Victoria Australia; ^5^ Epilepsy Research Centre, Department of Medicine University of Melbourne, Austin Health Heidelberg Victoria Australia; ^6^ Florey Institute of Neuroscience and Mental Health Parkville Victoria Australia

## Abstract

**Aim:**

To describe the phenotypic spectrum associated with stuttering.

**Method:**

Individuals with current or resolved developmental stuttering self‐referred. Surveys assessed stuttering characteristics (onset, negative impact, family history) and health (early development, other conditions). Speech and non‐verbal intelligence were assessed using conversation and the Wechsler standardized scales. Sample sizes varied across assessments (*n* = 266–327). Latent class analysis identified unobserved groups based on assessment data.

**Results:**

A total of 327 participants (231 male, 71%) with a median age of 57 years (range: 5–90 years) were recruited, 282 of 296 (95%) with current stuttering and 14 of 296 (5%) with resolved stuttering. Onset was 4 years or younger for 187 of 322 (58%) participants; 207 of 325 (64%) had a positive family history of stuttering, 58 of 325 (18%) had developmental delay, and 38 of 264 (14%) had below average non‐verbal intelligence. Common co‐occurring conditions included sleep, hearing, vision, and immune conditions, migraine, anxiety, and depression. Analysis revealed two groups: 295 of 327 (90%) participants had the common phenotype and 32 of 327 (10%) had a complex phenotype, with more severe stuttering, greater negative impact of stuttering, more frequent anxiety, lower non‐verbal intelligence, and neurodevelopmental disorders.

**Interpretation:**

Phenotypic analysis of a large cohort of who stutter identified 90% with a common phenotype and 10% with a complex phenotype. Both had co‐occurring disorders requiring multidisciplinary support.

## Graphical abstract

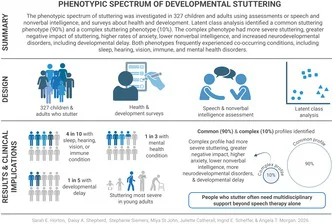

**Abbreviations**
PWS: people who stutter
LCA: latent class analysis
OASES: Overall Assessment of the Speaker's Experience of Stuttering

**What this paper adds**

- Developmental stuttering presents with common (90%) and complex (10%) phenotypes.
- Complex phenotypes were associated with neurodevelopmental disorders, anxiety, and more severe stuttering.
- Forty per cent of individuals reported sleep disturbance, hearing, vision, or immune disorders.
- Thirty‐three per cent of individuals had a mental health condition, frequently anxiety or depression.
- Stuttering was most severe in young adults, with moderate psychosocial impact.

Developmental stuttering is a neurodevelopmental disorder characterized by involuntary interruptions to speech, including repetitions, prolongations, and blocks with no sound, often accompanied by secondary tic‐like motor movements of the face and limbs.^1^ Increasingly, definitions include feelings of anticipation, loss of control, speech‐related anxiety, and avoidance behaviours.^1^, ^2^ Stuttering affects more than 10% of children between the ages of 2 years and 4 years but resolves in most cases, with adult prevalence estimated at approximately 1%.^3^, ^4^ Persistent stuttering into adolescence and adulthood is associated with reduced educational and vocational achievement,^5^ and elevated rates of anxiety disorders and social phobia.^6^

The cause of stuttering is unknown; however, there is a strong genetic predilection. Stuttering is highly heritable, with a positive family history reported in more than 50% of people who stutter (PWS).^7^ A small number of inherited and de novo pathogenic variants have been identified in either large families of PWS^8^, ^9^, ^10^ or in parent–child trios.^11^ Complex inheritance because of multiple genes of small effect underlies most cases.

Neuroimaging findings show structural and functional differences in brain regions governing speech timing and motor control, most notably the corticobasal ganglia–thalamocortical network.^12^ These regions have been implicated in human and mouse models with the pathogenic *PPID* variant,^8^ supporting theories that genetic and neurobiological factors interact and contribute to stuttering susceptibility.

PWS are heterogeneous, with varied symptomatology and common co‐occurring conditions. Children who stutter are 5.5 times more likely to have neurodevelopmental disorders such as intellectual disability, epilepsy, autism spectrum disorder, and attention‐deficit/hyperactivity disorder (ADHD) than typically fluent peers.^13^ Increased rates of neurodevelopmental, psychiatric, and other health conditions have also been reported in adults who stutter.^14^, ^15^ Children who stutter with co‐occurring conditions such as ADHD or developmental language disorder show poorer speech outcomes and greater socioemotional difficulties than those with stuttering alone.^16^, ^17^, ^18^

The striking heterogeneity in presentation and long‐term trajectory of stuttering has led to considerable interest in differentiating milder features, both in stuttering severity and likelihood of resolution. Small‐scale studies of stuttering characteristics, such as repetitions versus blocks, failed to find differentiating features.^19^, ^20^ Recent epidemiological studies, without direct speech assessment, highlight that stuttering is associated with other health conditions.^15^, ^16^ More fine‐grained phenotyping may assist with differentiating groups of PWS with the potential to guide clinical practice.

Our study aimed to describe the phenotypic spectrum of children and adults who stutter. We hypothesized that stuttering characteristics (severity, family history, age at onset), neurodevelopmental features, and co‐occurring conditions would contribute to stuttering phenotypes.

## METHOD

### Participants

We recruited participants from Australia and New Zealand enrolled in a genome‐wide association study of stuttering (beginning April 2018; see Boyce et al.^5^ for the methodology). Participants were aged 5 years or older, with a history of developmental stuttering (current or resolved). Cases of acquired stuttering were excluded.

This study was approved by the Human Research Ethics Committee at the Royal Children's Hospital, Melbourne (no. 37353) and the Health and Disability Ethics Committee in New Zealand (no. 20/CEN/63). Electronic informed consent was obtained from participants or caregivers.

### Data collection

Participants or caregivers previously completed a ‘core health survey’ regarding stuttering history and co‐occurring health conditions (Appendix S1).^5^ For this study, participants aged 18 years and older completed the online surveys. Where individuals were younger than 18 years, their caregivers completed the online surveys. All surveys were completed using the REDCap electronic data capture tools (hosted at Murdoch Children's Research Institute).^21^, ^22^ Participants attended a videoconference assessment between October 2021 and October 2024.

#### Online surveys

Participants or caregivers completed a health and development survey assessing early development (communication and motor milestones, regression, or loss of skills) and co‐occurring conditions (Appendix S2). Diagnoses of health conditions were assessed using a previously published survey (Appendix S1),^5^ which included the question ‘Have you ever been told by a doctor or health professional that you have any of the following conditions?’ and a checkbox list of conditions. Conditions were then grouped into domains, including neurological (e.g. movement disorder, epilepsy), immune system (e.g. allergies, asthma), sleep, mental health (e.g. anxiety disorder, affective disorder), neurodevelopmental (e.g. learning difficulties, ADHD), cardiovascular, musculoskeletal (e.g. arthritis), gut/digestive system (e.g. stomach ulcers), and endocrine system (e.g. diabetes).

To assess the negative impact of stuttering, participants or caregivers completed the age‐appropriate version of the standardized Overall Assessment of the Speaker's Experience of Stuttering (OASES),^23^, ^24^ which contains 60 to 100 items depending on the version. The OASES survey assesses impact across the general information, reactions to stuttering, communication in daily situations, and quality of life domains, and overall impact (total score). Higher scores reflect a more negative impact of stuttering.

#### Videoconference assessment

##### Stuttering assessment

Four speech pathologists conducted telehealth speech assessments (protocol shown in Appendix S3). Stuttering was assessed during 5 to 10 minutes of conversation. The speech pathologist recorded primary stuttering behaviours (e.g. blocks, repetitions of sounds) and secondary behaviours (e.g. rapid eye blinking), and rated stuttering severity on a 10‐point scale from 1 = no stuttering to 10 = extremely severe stuttering.^3^ Participants and caregivers rated stuttering severity using the same scale and reported any covert strategies (e.g. avoiding words or sounds). The reliability of videoconferencing to assess stuttering has been demonstrated for children and adults.^25^, ^26^, ^27^

The interrater reliability of stuttering ratings was investigated in a separate study using 154 conversation samples (52% of participants from this study). Pairs of ratings were highly correlated (*r* = 0.89, *p* <0.001).^28^

##### Family history interview

Speech pathologists interviewed participants or caregivers about a family history of stuttering, recording responses using standardized pedigree nomenclature.^29^

##### Assessment of co‐occurring speech disorders

Speech pathologists completed online ratings of features of co‐occurring (non‐stuttering) speech disorders based on oral motor and speech tasks, including the Oral and Speech Motor Control Protocol^30^—an assessment of oral anatomy (structure) and oral motor skill (function)—and a battery of speaking tasks increasing in motoric and cognitive complexity (Appendix S3). Tasks took approximately 15 minutes to complete. Videoconference is reliable for perceptual speech assessment, including speech sound errors, intelligibility, and oromotor function in children,^31^, ^32^, ^33^ and dysarthria and apraxia in adults.^27^, ^34^, ^35^

Co‐occurring speech disorders were classified as developmentally common (i.e. articulation and phonological disorders) or developmentally uncommon (i.e. motor speech disorders, including dysarthria and apraxia of speech). Any articulation or phonological errors were coded as age‐appropriate (e.g. resembling typical development), delayed or persistent, or atypical (e.g. the phonological error ‘fum’ for ‘thumb’ is age‐appropriate for a 5‐year‐old but delayed for a 10‐year‐old, while initial consonant deletion is an atypical error, occurring in less than 10% of children at any age^36^). The oral motor and speech tasks elicited sufficient range and complexity of speech and non‐speech tasks for speech pathologists to identify features of motor speech disorders (e.g. prosodic errors, reduced oral motor coordination). If present, features of motor speech disorders were rated using clinical norms and standardized rating scales,^37^, ^38^ and then coded as age‐appropriate (e.g. resembling normal ageing), mild, or moderate‐to‐severe. Perceptual evaluation combined with clinical judgement continues to be regarded as the best evidence‐based approach for assessing the presence and severity of motor speech disorders.^37^ To address overlap with primary stuttering behaviours, repetition and prolongation errors were excluded from the apraxia rating scale.^38^

##### Assessment of non‐verbal intelligence

Non‐verbal intelligence was assessed during videoconference assessment using the Wechsler Intelligence Scales, Fifth Edition Matrix Reasoning subtest (for 6–16 years,^39^ Wechsler Intelligence Scales for 16 years and older^40^). This subtest typically takes 5 to 10 minutes to complete. Participants viewed incomplete patterns and identified the missing piece from five options. Scaled scores were calculated using guidelines (<8 = below average, 8–12 = average, >12 = above average).

### Statistical analysis

Participant demographics were summarized (age, sex, main language). Stuttering severity (median, interquartile range [IQR]) and subjective impact of stuttering (mean, SD) were reported and visualized using Minitab v21.4 (Minitab LLC, State College, PA, USA) and Prism v10 (GraphPad Software, San Diego, CA, USA) stratified according to age.

Analysis was conducted on the available data, with missing data quantified and reported where appropriate. Latent class analysis (LCA) was used to investigate unobserved classes of PWS based on patterns across seven indicator variables, including: (1) a family history of stuttering (positive, negative); (2) age at stuttering onset (≤4 years, 5–8 years, ≥9 years); (3) stuttering presence and severity (resolved, mild, severity rating 1–3; moderate‐to‐severe, severity rating 4–10); (4) co‐occurring speech disorders (none, mild or resolved, severe or persistent); (5) development in early life (typical, mild or resolved delay, severe or persistent delay); (6) non‐verbal intelligence (average or above average, below average); and (7) co‐occurring health conditions (none, diagnoses in 1–3 health domains, diagnoses in ≥4 domains). LCA is a descriptive, person‐centred approach, chosen because it does not assume homogeneity in the population in question, unlike variable‐centred approaches. These indicator variables were chosen based on clinically relevant characteristics of interest that were potentially meaningful to differentiate latent classes (see Table S1 for the description and rationales). Before conducting the LCA, Spearman's rank correlations were estimated to ensure there was no high correlation between indicator variables.

LCA was conducted using Stata v18.0 (StatCorp, College Station, TX, USA) using the three‐step approach^41^ with all participants (*n* = 327). An optimal class number between two and four was selected using bootstrapped likelihood ratio tests and Bayesian and Akaike Information Criterion goodness‐of‐fit statistics (lower values indicate a better model fit, with Bayesian preferred for small sample sizes <300).^42^ Classes were capped at four to ensure that participant subgroups would be clinically interpretable. Entropy (range: 0–1) assessed classification accuracy, with values greater than 0.80 indicating good separation between classes.^42^ After the best model was chosen, posterior probabilities (range: 0–1) were used to assign participants to the most probable class, with higher values reflecting more accurate classification. Age and the negative impact of stuttering were introduced retrospectively as inactive variables of interest. The characteristics of each group were described and compared (mean differences for continuous variables; odds ratios [ORs] to estimate the likelihood of trait between groups).

As sensitivity testing, LCA was repeated to assess the stability of latent classes for different analytical samples: (1) retaining only participants with persistent stuttering (*n* = 313), considering that those with resolved stuttering may have different characteristics to those with persistent stuttering; (2) retaining only complete cases (*n* = 260), considering that missing data may influence the resulting latent classes; (3) excluding the youngest group of participants (<15 years; *n* = 295), considering that age may influence the resulting latent classes; and (4) with all participants recruited and including age as a covariate (*n* = 327), considering that age may influence the resulting latent classes. Age distribution was also described across LCA classes for all analyses.

## RESULTS

### Participant demographics

A total of 1430 participants were invited and 330 (23%) took part; 299 of 330 (91%) completed a videoconference speech assessment, 282 of 330 (86%) completed the health and development survey, and 271 of 330 (82%) completed the OASES. Three adults were excluded after assessment because of acquired stuttering or neurological events affecting speech evaluation (Figure S1), resulting in an analytical sample of 327 participants (median age = 57 years, range: 5–90 years), including 27 children (5–12 years), 12 adolescents (13–17 years), and 288 adults (≥18 years; Table 1). Compared to individuals who were invited but did not participate or were lost to follow‐up (*n* = 1103), the analytical sample (*n* = 327) had a similar proportion of male participants (71% compared with 67%), more adults than children (88% compared with 82%), more retirees (23% compared with 15%), higher educational attainment (28% with a postgraduate degree compared with 19%), and higher median salary (AUS$90 k compared with AUS$80 k for full time employees; see Table S2 for all comparisons).

**Table 1:** Participant demographics and stuttering classification, stratified according to age.

Variable	Total (*n* = 327)	Age, years							
		5–14 (*n* = 32)	15–24 (*n* = 14)	25–34 (*n* = 31)	35–44 (*n* = 33)	45–54 (*n* = 41)	55–64 (*n* = 45)	65–74 (*n* = 85)	≥ 75 (*n* = 46)
Sex									
Male	231 (71)	24 (75)	10 (71)	17 (55)	25 (76)	25 (61)	31 (69)	64 (75)	35 (76)
Female	96 (29)	8 (25)	4 (29)	14 (45)	8 (24)	16 (39)	14 (31)	21 (25)	11 (24)
Main language									
English	312/322 (97)	29/29 (100)	12/14 (86)	27/31 (87)	31/33 (94)	40/41 (98)	42/43 (98)	85/85 (100)	46/46 (100)
Other	10/322 (3)	N/A	2/14 (14)	4/31 (13)	2/33 (6)	1/41 (2)	1/43 (2)	N/A	N/A
Resolved or persistent stuttering^a^ (based on speech assessment with *n* = 296)									
Resolved	14/296 (5)	1/29 (3)	1/12 (8)	3/28 (11)	2/27 (7)	2/39 (5)	1/42 (2)	3/81 (4)	1/38 (3)
Persistent	282/296 (95)	28/29^b^ (97)	11/12 (92)	25/28 (89)	25/27 (93)	37/39 (95)	41/42 (98)	78/81 (96)	37/38 (97)

*Note*: Data are *n* (%). Other main languages listed: Mandarin, Russian, Mauritian Creole/French, Nepali, Tamil, Thai, Croatian, Hindi, Italian. Abbreviation: N/A, not applicable.

^a^ Resolved stuttering indicates that the participant reported that their stuttering had resolved, and stuttering severity was rated as 1 (no stuttering) during speech assessment by both clinician and participant. Persistent stuttering indicates a stuttering severity greater than 1 during speech assessment or the participant self‐reported that they still stuttered (not observed during assessment).

^b^ Stuttering was considered ‘current’ rather than ‘persistent’ in *n* = 27 children younger than 13 years because of frequent spontaneous resolution in this age group.

### Summary of indicator variables

**Figure 1:**
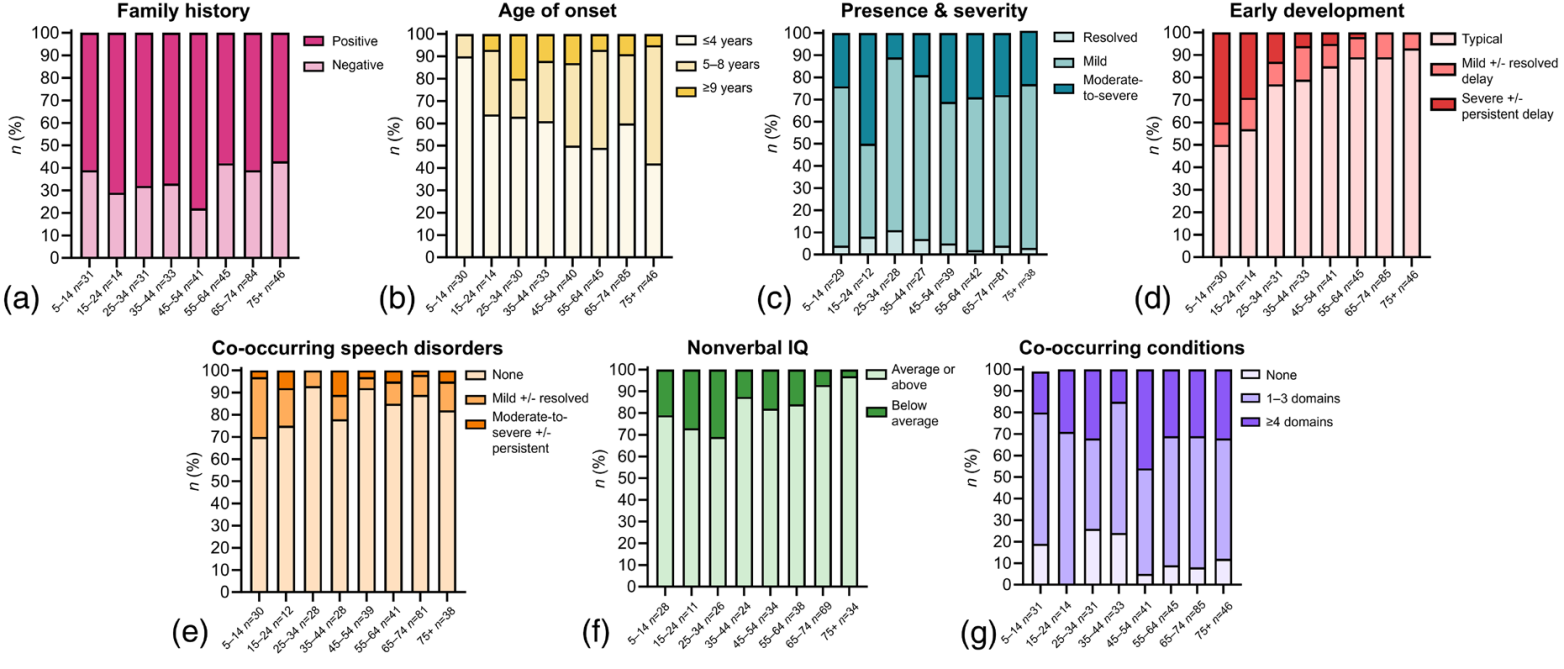
Participants' indicator variable classifications, stratified according to age (years). (a) Family history of stuttering. (b) Age at stuttering onset. (c) Stuttering presence and severity. (d) Development in early life. (e) Features of co‐occurring speech disorders. (f) Non‐verbal intelligence. (g) Co‐occurring health conditions. The stacked bars represent the percentage of participants classified into corresponding response category. Age groups from left to right: 5–14 years, 15–24 years, 25–34 years, 35–44 years, 45–54 years, 55–64 years, 65–74 years, 75+ years.

Participants were classified into indicator variable response categories using survey and assessment data (Table S1) and described according to age group (Figure 1).

#### Stuttering phenotype

**Figure 2:**
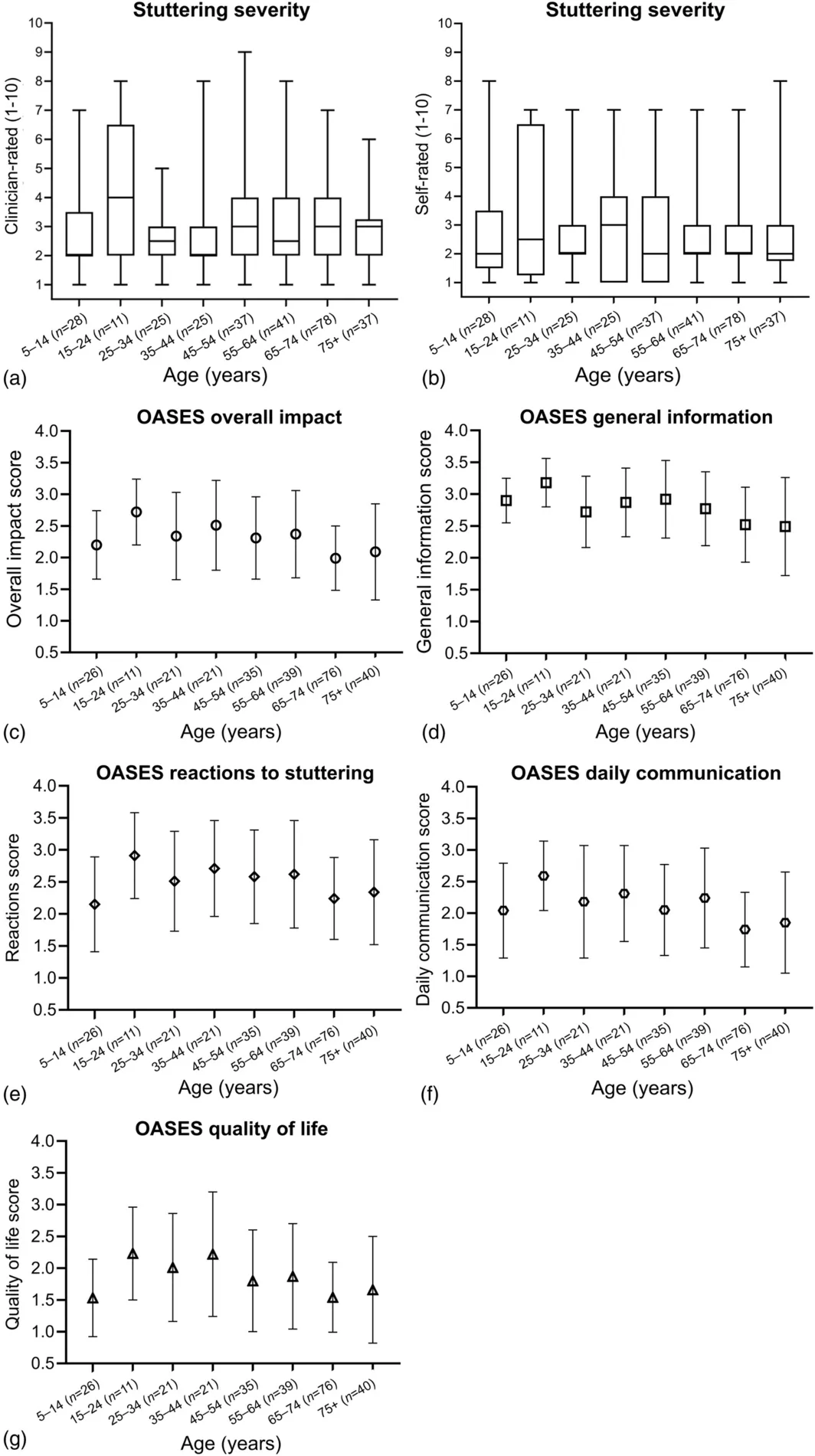
Stuttering measures according to age. (a) Clinician‐rated stuttering severity. (b) Participant self‐rated stuttering severity. (c) Overall Assessment of the Speaker's Experience of Stuttering (OASES) overall impact score. (d–g) OASES subdomain scores: general information (d), reactions to stuttering (e), communication in daily situations (f), quality of life (g). (a,b) The boxes represent the interquartile range, the error bars represent the total range, and the horizontal line represents the median. Stuttering severity scale: 1 = no stuttering, 10 = extremely severe stuttering. (c–g) Symbols represent the group mean, the error bars represent the SD.

In total, 207 of 325 (64%) participants reported a positive family history of stuttering; 187 of 322 (58%) had stuttering onset at age 4 years or younger, 106 of 322 (33%) between 5 years and 8 years, and 29 of 322 (9%) at age 9 years or older. A total of 296 participants completed speech assessment; 27 (9%) were younger than 13 years and too young to classify as resolved or persistent because of high rates of spontaneous resolution at this age.^43^ Of the remaining 269, only 14 (5%) had resolved stuttering confirmed by self‐report and speech assessment (seven resolved less than 9 years and seven resolved 9 years or more) and 255 of 269 (95%) had persistent stuttering. In 255 with 13 or more years of persistent stuttering, 24 of 255 (9%) did not display overt stuttering during speech assessment (severity = 1) but reported persistent stuttering, 163 of 255 (64%) had mild stuttering (severity = 2–3), and 68 of 255 (27%) had moderate‐to‐severe stuttering (severity = 4–10). Median stuttering severity was 2 to 3 for self‐ratings and clinician‐ratings across all age groups, except young adults (15–24 years), whose median clinician rating was 4. The mean negative stuttering impact (OASES score) showed a similar pattern, with mild‐to‐moderate impact (mean = 2.22, SD = 0.65) across all ages; however, young adults were again more severe, with a moderate impact (mean = 2.70, SD = 0.52). Scores trended downwards for older ages, with the lowest scores for 65‐ to 74‐year‐olds (mean = 1.99, SD = 0.51; Figure 2).

In 282 participants with persistent stuttering, sound repetitions were most frequent (214 of 282, 76%), followed by blocks (177 of 282, 63%), and word repetitions (121 of 282, 43%). The most frequent secondary behaviours included facial grimacing (52 of 282, 18%) and rapid eye blinking (45 of 282, 16%). Stuttering behaviours were similar across age groups; however, our small cohort of young adults demonstrated more frequent secondary behaviours, which is consistent with more severe stuttering overall (Figure S2). Some participants reported using covert strategies to control stuttering, including word and sound avoidance (47 of 282, 17%), and changing word order (30 of 282, 11%; Table S3).

#### Features of co‐occurring speech disorders

A total of 296 participants completed a speech assessment; 252 of 296 (85%) showed no evidence of atypical speech features beyond stuttering; 33 of 296 (11%) had a mild or resolved speech disorder because of (1) history of articulation or phonological disorder (*n* = 15) or (2) articulation or phonological errors observed during assessment (*n* = 22); 12 of 296 (4%) had a severe or persistent speech disorder because of (1) diagnosis of apraxia of speech (*n* = 1) or (2) features of these disorders observed during assessment (*n* = 12; Table S4). One participant had cluttering, a fluency disorder characterized by irregular speech rate, coarticulation, and excessive ‘typical’ disfluencies such as ‘um’.

#### Early development

A total of 267 of 325 (82%) participants reported typical development in early life or no known delay, while 33 of 325 (10%) reported isolated delays in communication or motor milestones, and 25 of 325 (8%) reported delays across both communication and motor milestones or caregiver concern regarding loss of skills (Table S5). Mild or resolved and severe or persistent developmental delays were most frequently reported in 5‐ to 14‐year‐olds (15 of 30, 50%), with the fewest cases in the oldest age group (≥75 years; 3 of 46, 7%; Figure 1).

#### Non‐verbal intelligence

Half of participants (131 of 264, 50%) received average scores for the Matrix Reasoning test, while 95 of 264 (36%) scored above average, and 38 of 264 (14%) below average (>1SD above or below the mean).

#### Co‐occurring health conditions

Most participants (183 of 326, 56%) reported co‐occurring conditions in 1 to 3 health domains; 39 of 326 (12%) reported no conditions and 104 of 326 (32%) reported conditions in 4 or more domains. The median co‐occurring conditions was 3.5 (IQR = 2–6), across a median of 3 health domains (IQR = 1–4), which was broadly similar across age groups.

We compared the frequency of co‐occurring conditions in our sample to the population prevalence rates (Table 2). Of note, more than 40% reported sleep disturbance, an immune system condition, or a neurological condition, and one‐third (33%) reported a mental health condition. Conditions with higher frequency compared to the population prevalence were described according to age (Figure S3).

**Table 2:** Summary of participant‐reported health conditions and population prevalence estimates, according to health domain.

Health domain and diagnosis	Frequency in sample		Population prevalence estimate
	*n*/total	%	%
Sleep ≥1 diagnosis	122/280	43.6	–
Sleep disturbance, including insomnia, sleep apnoea, frequent waking	122/280	43.6^a^	23.8
(Free text) Narcolepsy	2/323	0.9	0.01–0.07
Neurological ≥1 diagnosis	136/326	41.7	–
Hearing disorders	63/326	19.3^a^	9.6
Vision disorders (cannot be corrected by glasses or contact lenses)	53/326	16.3^a^	3.8
Migraine	38/326	11.6^a^	6.6
Movement disorder	17/280	6.1	–
Brain injury or concussion	19/326	5.8	2–5
Epilepsy or seizure disorder	6/326	1.8	0.2
Stroke	7/326	2.1	0.8–1.6
Memory problem	4/326	1.2	1.5
Immune system ≥1 diagnosis	135/326	41.4	–
Allergies, including hay fever, seasonal allergies, food allergies	85/326	26.1	14.0–23.9
Asthma	63/326	19.3^a^	10.8
Skin problems, including dermatitis, eczema, psoriasis	50/280	17.9^a^	4.1–4.9
Blood or immune system problems, other immune disorder	11/280	3.9	~4%
Mental health ≥1 diagnosis	108/326	33.1	–
Anxiety or panic disorder	91/326	27.8^a^	18.9
Affective disorder, including depression, depressive disorder, manic depression, bipolar disorder	67/326	20.5	12.8
Posttraumatic stress disorder	14/326	4.3	2.6
Eating disorder, including anorexia, bulimia	9/326	2.8	<1.0–8.4
Alcohol or other substance misuse, drug addiction	6/287	1.8	0.7
Obsessive‐compulsive disorder	4/323	1.2	1.9
Schizophrenia, psychosis	2/323	0.6	0.5
Premenstrual dysphoric disorder	1/323	0.3	1.6
(Free text response) Selective mutism	1/326	0.3	0.7
Musculoskeletal system ≥1 diagnosis	80/326	24.5	–
Arthritis or rheumatism	67/326	20.5	14.5
Other musculoskeletal problems	30/326	9.2	3.1
Cardiovascular system ≥1 diagnosis	68/326	20.9	5.5
Heart attack or disease	49/326	15.0^a^	2.3
Hypertension or high blood pressure	34/323	10.5	11.6
Neurodevelopment ≥1 diagnosis	66/326	20.2	–
Attention‐deficit/hyperactivity disorder, attention, or concentration problems	41/326	12.5^a^	~5.4–7.5
Learning difficulties	22/326	6.7	~8
Global developmental delay	17/326	5.2	1–3
Behaviour or conduct problems, oppositional disorder	17/326	5.2	1.6–2.5
Autism spectrum disorder, Asperger	14/326	4.3	1.1
Language disorder, including expressive, receptive, semantic, or grammar disorder	12/323	4.5	6.4
Reading or spelling disorder, including dyslexia	12/323	4.5	10
Intellectual disability	10/326	3.1	1–2
Sensory processing disorder	4/280	1.2	~5.0–6.5
Auditory processing disorder	4/280	1.2	~5.0–6.5
Developmental coordination disorder	1/280	0.4	5.0
Speech ≥1 diagnosis	16/323	5.0	–
Articulation or phonological disorder	15/323	4.6	8–9
Apraxia of speech	1/323	0.3	0.1
Dysarthria	0/323	0	0.1
Gut or digestive system ≥1 diagnosis	38/326	11.7	7.1
Problems with appetite or digestion	33/326	10.1	–
Stomach ulcers	7/323	2.2	1.6
(Free text) Oesophageal problems or reflux	5/326	1.5	1.7
Endocrine system ≥1 diagnosis	33/326	10.1	–
Total endocrine, nutritional, and metabolic diseases (including high cholesterol)	37/326	11.3	17.4
Diabetes (any)	18/326	5.5	5.3
Problems with thermal regulation	9/326	2.8	–
Endocrine disorder, metabolic system problems	5/326	1.5	2.6
Early onset of puberty	3/326	0.9	7–16
Problems with growth or short stature	2/280	0.7	~2.5
Other conditions			
Vision disorders that can be corrected by glasses or contact lenses	158/280	56.4	~50
Total vision diseases of the eye and adnexa	162/326	49.7	56.7
Neck or back problems	29/323	8.9	15.7
Cancer (any)	21/323	6.4	2.3
Chronic pain	15/323	4.6	<10–31
Renal, kidney, bladder, or urogenital system problems	15/326	4.6	2.7
Chronic fatigue	3/326	0.9	~0.7
Chronic lung disease	4/323	1.2	0.4–2.5
(Free text) High cholesterol	5/326	1.5	8.4
(Free text) Cleft palate or congenital abnormalities	1/326	0.3	0.8
(Free text) Genetic diagnosis	1/326	0.3	0.8
Health domains (Australian Bureau of Statistics classifications)			
Intellectual disability^b^	32/326	9.8	1.2–5.2
Mental and substance use problems^c^	136/326	41.7	25.9
Vision or hearing disorder^d^	99/326	30.4	21.3
Neurological problems^e^	67/326	20.6	8.8
Musculoskeletal disorders^f^	96/326	29.5	28.7

*Note*: The diagnosis indicates that the participant reported receiving a diagnosis from a doctor or health professional. (Free text) refers to conditions not listed in the survey but specified by the participant(s). Population prevalence estimates are based on Australian Bureau of Statistics data, where available.

^a^ Frequency in more than 50% of age groups was higher than expected compared to the population prevalence data. A dash indicates that no equivalent data were available.

^b^ Defined as ‘difficulty learning or understanding things’, including learning difficulties, intellectual disability, reading, or spelling disorder.

^c^ Includes neurodevelopmental disorders (e.g. attention‐deficit/hyperactivity disorder, autism spectrum disorders, intellectual disability, learning difficulties, speech or language disorders) and mental health disorders (e.g. anxiety disorders, posttraumatic stress disorder, affective disorders, substance use problems).

^d^ Excludes vision disorders corrected by glasses.

^e^ Excludes hearing and vision disorders; includes dementia, epilepsy, migraine, movement disorders, progressive neurological disorders (e.g. Parkinson disease), chronic fatigue syndrome, narcolepsy.

^f^ Includes arthritis, musculoskeletal and connective tissue conditions, back problems.

### Latent class analysis

The Spearman's rank correlations between indicator variables were small (ranging from −0.08 to 0.20), indicating negligible risk of redundancy among variables. The highest correlation was between development in early life and co‐occurring speech disorders (*r*_*s*_ = 0.20 [0.09–0.31]; Table S6 for correlation matrix). Bayesian and Akaike's values for the primary LCA and three of the four sensitivity tests indicated that a two‐class model was optimal, with entropy values reflecting adequate separation between classes (Table 3). Sensitivity tests restricted to participants with persistent stuttering (1; *n* = 313), complete cases (2; *n* = 260), and with the youngest participants excluded (3; *n* = 295) showed similar results to the primary analysis that included all participants (*n* = 327; Table 3 and Tables S7, S8, and S9). Sensitivity test 4, including all cases and age as a covariate (*n* = 327), indicated that a three‐class model was optimal; however, including age in the model altered class composition such that classes primarily reflected age differences (Table S10). We determined that age was driving the model and thus pulled away from the interpretation of other variables of primary interest. As such, the original model with all participants and indicator variables of interest, excluding age, was retained as our primary focus for all further analyses.

**Table 3:** Latent class analysis model fit statistics.

	Number of classes	Bayesian information criterion	Akaike's information criterion	Entropy
All cases *n* = 327	2	2852.66	2765.49	0.79
	3	2902.39	2777.32	0.38
	4	2931.17	2779.57	0.37
Sensitivity test 1: cases with resolved stuttering excluded *n* = 313	2	2653.17	2574.50	0.83
	3	2582.12	2702.00	0.60
	4	2721.2	2586.33	0.34
Sensitivity test 2: complete cases *n* = 260	2	2355.41	2273.52	0.72
	3	2397.55	2276.49	0.66
	4	2447.39	2287.16	0.44
Sensitivity test 3: cases with the youngest group (<15 years) excluded *n* = 295	2	2539.33	2454.53	0.80
	3	2580.08	2462.10	0.38
	4	2611.33	2463.85	0.39
Sensitivity test 4: all cases, with age as a covariate *n* = 327	2	2809.22	2718.26	0.75
	3	2837.61	2697.38	0.86
	4	2708.88	2894.59	0.59

*Note*: ‘All cases’ was used as the final analytical sample.

#### Class descriptions

**Figure 3:**
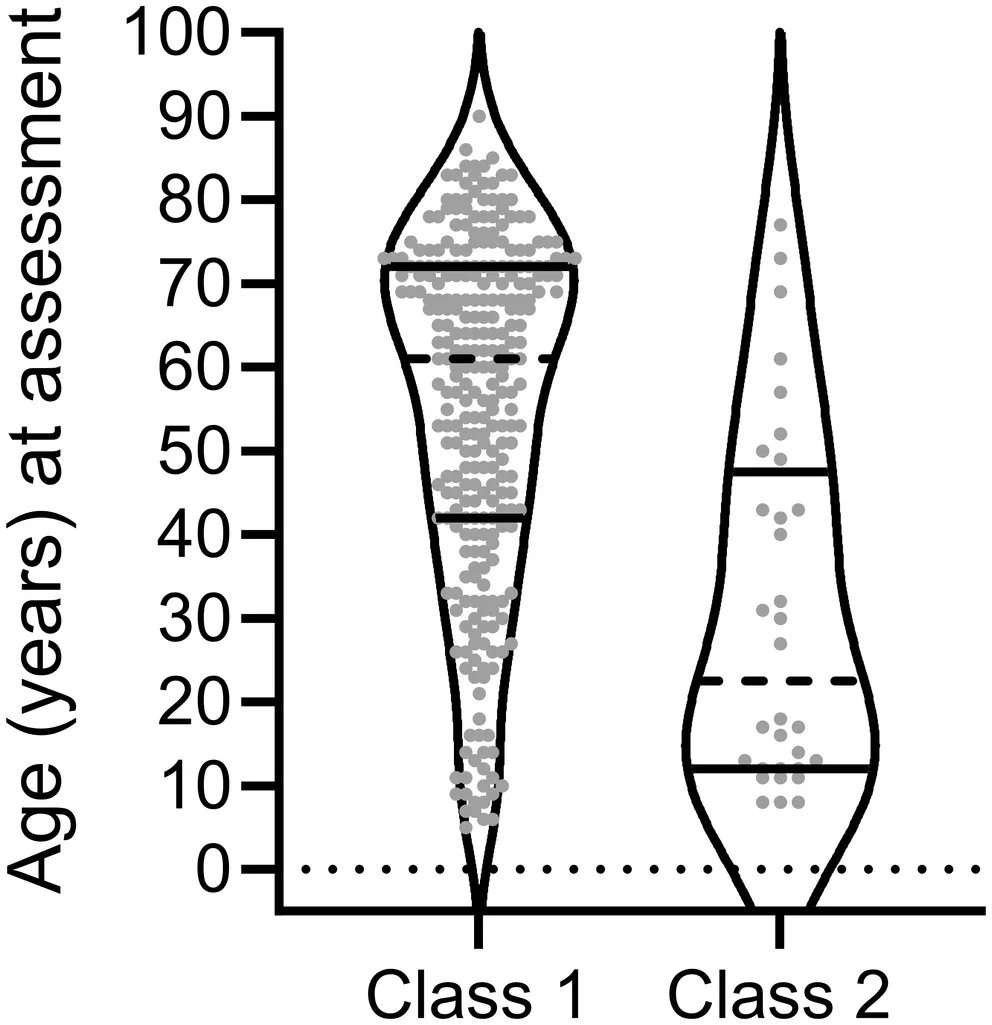
Participant age at assessment according to latent class. The dashed line represents median, the solid lines represent the interquartile range, and the dots represent individual participant data.

**Figure 4:**
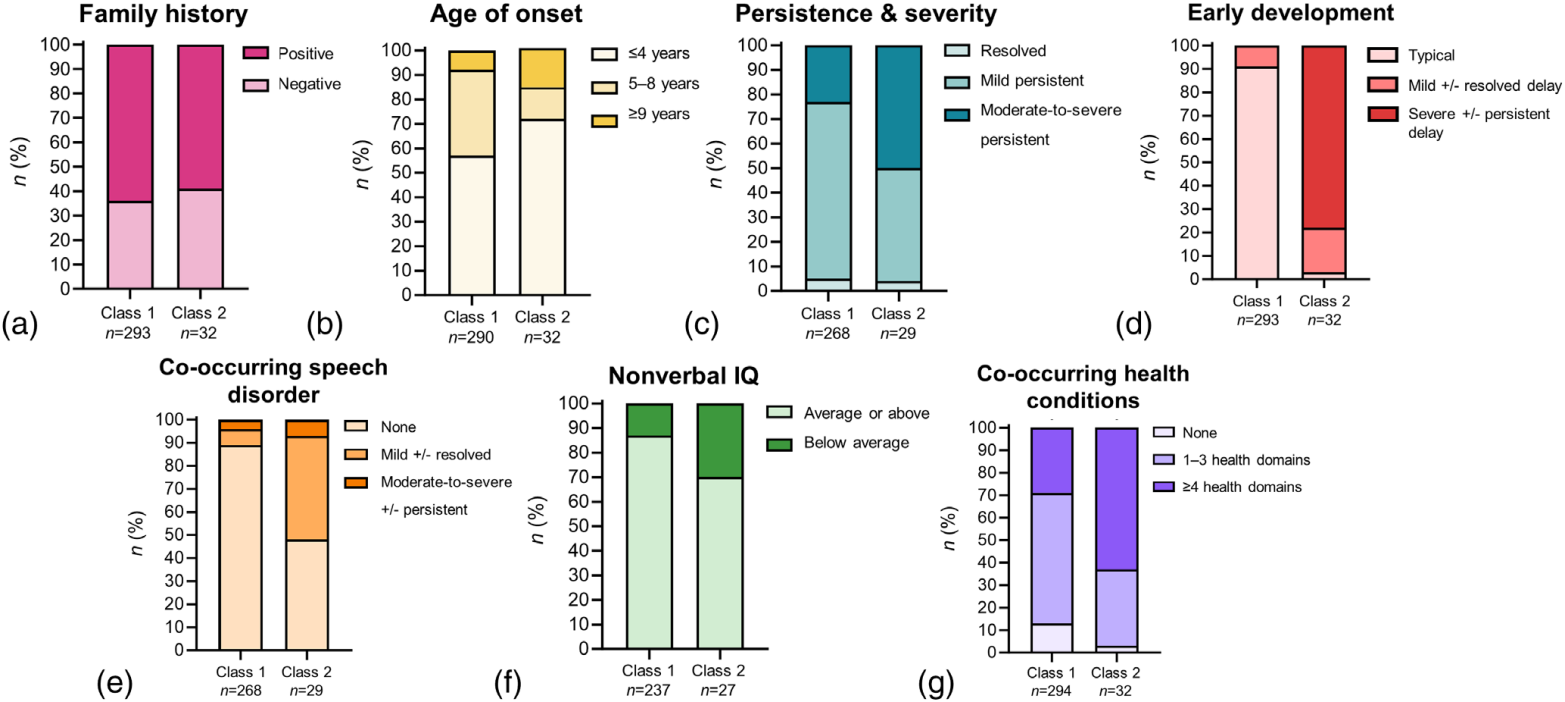
Participants' indicator variable classifications, stratified according to latent class membership. (a) Family history of stuttering. (b) Age at stuttering onset. (c) Stuttering presence and severity. (d) Development in early life. (e) Features of co‐occurring speech disorders. (f) Non‐verbal intelligence. (g) Co‐occurring health conditions.

Classes 1 and 2 included 90% (*n* = 295) and 10% (*n* = 32) of participants respectively. Male sex was distributed similarly between classes 1 (71%) and 2 (66%). Class 1 was older (mean = 55 years, SD = 21, range: 5–90) than class 2 on average (mean = 31 years, SD = 22, range: 8–77; mean difference = 24 years [14 years 11 months–32 years 10 months]; Figure 3). Classes 1 and 2 were the most different for the indicator variables of early development and co‐occurring speech and health conditions, with class 2 being more severe. Differences were less marked between classes for non‐verbal intelligence, stuttering onset, and stuttering severity (Figure 4).

#### Stuttering phenotype

A positive family history was similar for classes 1 (64%) and 2 (59%). Class 2 had higher odds than class 1 of both earlier stuttering onset (≤4 years; OR = 2.38 [0.99–5.69]) and later onset (≥9 years; OR = 3.18 [1.12–9.06]), whereas class 1 had more individuals with middle‐range onset (5–8 years; OR = 8.86 [2.20–35.69]; Table 4). Resolved stuttering was similar between classes; however, class 2 had lower odds of mild stuttering (OR = 0.29 [0.13–0.68]) and greater odds of moderate‐to‐severe stuttering (OR = 4.10 [1.75–9.60]). Primary stuttering behaviours (e.g. repetitions, blocks) were similar between classes; however, secondary behaviours (e.g. eye blinking) were more frequent in class 2 (Table S11). OASES total scores were also higher for class 2 (mean = 2.68, SD = 0.53) than class 1 (mean = 2.17, SD = 0.65; mean difference = 0.51 [0.28–0.73]), indicating a more negative impact of stuttering.

**Table 4:** Frequency counts and odds ratios for indicator variable response categories, stratified according to latent class.

Indicator variable and response category	All *n*/327 (%)	Class 1 *n*/295 (%)	Class 2 *n*/32 (%)	Odds ratio (95% CI)	Interpretation of odds ratio: class 2 vs class 1
1. Family history of stuttering					
a: No family history	118/325 (36)	105/293 (36)	13/32 (41)	1.45 (0.66–3.18)	Higher odds of no family history
b: Positive family history	207/325 (64)	188/293 (64)	19/32 (59)	0.69 (0.31–1.51)	Lower odds of positive family history
2. Age at stuttering onset					
a: Onset ≤4 years	187/322 (58)	164/290 (57)	23/32 (72)	2.38 (0.99–5.69)	Higher odds of onset ≤4 years
b: Onset 5–8 years	106/322 (33)	102/290 (35)	4/32 (13)	0.11 (0.03–0.45)	Lower odds of onset 5–8 years
c: Onset ≥9 years	29/322 (9)	24/290 (8)	5/32 (16)	3.18 (1.12–9.06)	Higher odds of onset ≥9 years
3. Stuttering presence and severity					
a: Resolved	14/296 (5)	13/268 (5)	1/28 (4)	0.45 (0.04–5.83)	Lower odds of resolved stuttering
b: Mild	205/296 (69)	192/268 (72)	13/28 (46)	0.29 (0.13–0.68)	Lower odds of mild stuttering
c: Moderate‐to‐severe	77/296 (26)	63/268 (23)	14/28 (50)	4.10 (1.75–9.60)	Higher odds of moderate‐to‐severe stuttering
4. Development in early life					
a: Typical development	267/325 (82)	266/293 (91)	1/32 (3)	<0.01 (<0.01–0.01)	Lower odds of typical development
b: Mild or resolved developmental delay	33/325 (10)	27/293 (9)	6/32 (19)	5.35 (2.09–13.73)	Higher odds of mild or resolved delay
c: Severe or persistent delay/disorder	25/325 (8)	0/293 (0)	25/32 (78)	Unable to calculate	All cases classified to class 2
5. Co‐occurring speech disorder					
a: No other speech disorder	252/297 (85)	238/268 (89)	14/29 (48)	0.08 (0.03–0.19)	Lower odds of no other speech disorder
b: Mild or resolved speech disorder	33/297 (11)	20/268 (7)	13/29 (45)	12.77 (4.95–32.95)	Higher odds of mild or resolved speech disorder
c: Moderate‐to‐severe or persistent speech disorder	12/297 (4)	10/268 (4)	2/29 (7)	4.07 (0.94–17.54)	Higher odds of severe or persistent speech disorder
6. Non‐verbal intelligence					
a: Average or above average score	226/264 (86)	207/237 (87)	19/27 (70)	0.31 (0.12–0.82)	Lower odds of average or above average score
b: Below average score	38/264 (14)	30/237 (13)	8/27 (30)	3.25 (1.22–8.67)	Higher odds of below average score
7. Co‐occurring health conditions					
a: No other diagnoses	39/326 (12)	38/294 (13)	1/32 (3)	0.02 (<0.001–1.45)	Lower odds of no other diagnoses
b: Diagnoses in 1–3 health domains	183/326 (56)	172/294 (58)	11/32 (34)	0.31 (0.14–0.71)	Lower odds of diagnoses in 1–3 health domains
c: Diagnoses in ≥4 health domains	104/326 (32)	84/294 (29)	20/32 (63)	6.35 (2.75–14.69)	Higher odds of diagnoses in ≥4 health domains
Total	326	294	32		

*Note*: The odds ratios were calculated using binary logistic regression analysis with probability of class 2 membership as a continuous variable (range: 0–1). We were unable to calculate some odds ratios because of complete separation of data between classes. Sample sizes for individual measures ranged from *n* = 264 to 326. Abbreviation: CI, confidence interval.

#### Co‐occurring features

Mild or resolved (non‐stuttering) speech disorders were more frequent in class 2 than class 1 (OR = 12.77 [4.95–32.95]). In terms of developmental delay, the model assigned all participants with severe or persistent developmental delay (*n* = 25) to class 2. Comparatively, most of class 1 (*n* = 266, 91%) reported typical development and 9% (*n* = 27) reported a mild or resolved delay. Class 2 was also more likely to score below average non‐verbal intelligence on the Matrix Reasoning test (OR = 3.25 [1.22–8.67]) than class 1.

Co‐occurring conditions were investigated according to class (Table 5). Class 2 reported more health conditions than class 1 (median = 8 [IQR = 4.25–11] and median = 3 [IQR = 1–5] respectively). Neurodevelopmental disorders were substantially higher for class 2 (OR = 89.73 [25.55–315.07]), as were sleep (OR = 7.21 [2.96–17.56]), mental health (OR = 3.31 [1.50–7.30]), and immune (OR = 2.71 [1.22–6.02]) conditions.

**Table 5:** Frequency counts and odds ratios for health conditions, stratified according to latent class.

Health domain and condition	All *n*/total (%)	Class 1 *n*/295	Class 2 *n*/32	Odds ratio (95% CI)
Neurodevelopmental ≥1 diagnosis	66/326 (20.2)	39 (13.2)	27 (84.4)	89.73 (25.55–315.07)
Language disorder	12/323 (4.5)	3 (1.0)	9 (28.1)	125.96 (17.54–904.78)
Learning difficulties	22/326 (6.7)	8 (2.7)	14 (43.8)	46.91 (15.36–143.22)
Autism spectrum disorder	14/326 (4.3)	5 (1.7)	9 (28.1)	40.17 (10.67–151.23)
Reading or spelling disorder	12/323 (4.5)	5 (1.7)	7 (21.9)	25.58 (3.40–94.44)
Behavioural or conduct disorder	17/326 (5.2)	9 (3.1)	8 (25.0)	14.13 (4.55–43.87)
Sensory or auditory processing disorder	4/280 (1.2)	2 (0.8)	2 (6.7)	11.82 (1.36–102.81)
Articulation or phonological disorder	15/323 (4.6)	9 (3.1)	6 (18.8)	11.59 (3.52–38.17)
Attention‐deficit/hyperactivity disorder	41/326 (12.5)	27 (9.2)	14 (43.8)	11.45 (4.78–27.56)
Global developmental delay	17/326 (5.2)	0 (0)	17 (53.1)	Unable to calculate
Intellectual disability	10/326 (3.1)	0 (0)	10 (31.3)	Unable to calculate
Sleep disturbance	122/280 (43.6)	99 (39.4)	23 (76.7)	7.21 (2.96–17.56)
Mental health ≥1 diagnosis	108/326 (33.1)	91 (30.8)	17 (53.1)	3.31 (1.50–7.30)
Anxiety disorder	91/326 (27.8)	75 (25.4)	16 (50.0)	4.05 (1.83–9.00)
Alcohol or other substance misuse, drug addiction	6/287 (1.8)	4 (1.4)	2 (6.3)	4.39 (0.62–30.92)
Posttraumatic stress disorder	14/326 (4.3)	11 (3.7)	3 (9.4)	3.56 (0.90–14.16)
Affective disorder	67/326 (20.5)	60 (20.3)	7 (21.9)	1.34 (0.54–3.32)
Eating disorder, including anorexia, bulimia	9/326 (2.8)	8 (2.7)	1 (3.1)	1.28 (0.14–11.40)
Premenstrual dysphoric disorder	1/323 (0.3)	1 (0.3)	1 (3.1)	12.01 (0.58–248.33)
Schizophrenia, psychosis	2/323 (0.6)	0 (0)	1 (3.1)	Unable to calculate
Obsessive‐compulsive disorder	4/323 (1.2)	4 (1.4)	0 (0)	Unable to calculate
(Free text) Selective mutism	1/326 (0.3)	0 (0)	1 (3.1)	Unable to calculate
Immune system ≥1 diagnosis	135/326 (41.4)	116 (39.3)	19 (59.4)	2.71 (1.22–6.02)
Skin problems, including dermatitis, eczema	50/280 (17.9)	39 (15.5)	11 (36.7)	3.99 (1.68–9.45)
Allergy	85/326 (26.1)	73 (24.7)	12 (37.5)	2.08 (0.92–4.67)
Blood or immune system problems	11/280 (3.9)	9 (3.6)	2 (6.7)	2.08 (0.37–11.77)
Asthma	63/326 (19.3)	54 (18.3)	9 (28.1)	2.02 (0.85–4.83)
Digestive system ≥1 diagnosis	38/326 (11.7)	31 (10.5)	7 (21.9)	2.68 (1.01–7.12)
Problems with appetite or digestion	33/326 (10.1)	26 (8.8)	7 (21.9)	3.34 (1.24–9.03)
Stomach ulcers	7/323 (2.2)	7 (2.4)	0 (0)	Unable to calculate
(Free text) Oesophageal problems or reflux	5/326 (1.5)	5 (1.7)	0 (0)	Unable to calculate
Endocrine system ≥1 diagnosis	33/326 (10.1)	27 (9.2)	6 (18.8)	2.62 (0.93–7.36)
Early onset of puberty	3/326 (0.9)	2 (0.7)	1 (3.1)	6.93 (0.54–88.92)
Problems with thermal regulation	9/326 (2.8)	7 (2.4)	2 (6.3)	3.88 (0.74–20.29)
Endocrine disorder, metabolic system problems	5/326 (1.5)	4 (1.4)	1 (3.1)	2.49 (0.23–27.48)
Diabetes (any)	18/326 (5.5)	16 (5.4)	2 (6.3)	1.03 (0.19–5.53)
Problems with growth or short stature	2/280 (0.7)	0 (0)	2 (6.7)	Unable to calculate
Neurological ≥1 diagnosis	136/326 (41.7)	119 (40.3)	17 (53.1)	1.65 (0.76–3.58)
Movement disorder	17/280 (6.1)	11 (4.4)	6 (20.0)	7.07 (2.21–22.63)
Epilepsy	6/326 (1.8)	5 (1.7)	1 (3.1)	2.70 (0.31–23.35)
Brain injury or concussion	19/326 (5.8)	16 (5.4)	3 (9.4)	1.85 (0.46–7.45)
Headache or migraine	38/326 (11.6)	32 (10.8)	6 (18.8)	1.78 (0.62–5.12)
Vision disorders (cannot be corrected by glasses or contact lenses)	53/326 (16.3)	46 (15.6)	7 (21.9)	1.51 (0.58–3.96)
Hearing disorder	63/326 (19.3)	57 (19.3)	5 (15.6)	0.64 (0.21–1.92)
Memory problem	4/326 (1.2)	4 (1.4)	0 (0)	Unable to calculate
Stroke	7/326 (2.1)	7 (2.4)	0 (0)	Unable to calculate
Cardiovascular system ≥1 diagnosis	68/326 (20.9)	62 (21.0)	6 (18.8)	0.87 (0.33–2.33)
Heart attack or disease	49/326 (15.0)	44 (14.9)	5 (15.6)	1.21 (0.43–3.41)
Hypertension or high blood pressure	34/326 (10.5)	32 (10.8)	2 (6.3)	0.49 (0.10–2.38)
Musculoskeletal system ≥1 diagnosis	80/326 (24.5)	75 (25.4)	5 (15.6)	0.64 (0.24–1.72)
Arthritis or rheumatism	67/326 (20.5)	64 (21.7)	3 (9.4)	0.42 (0.13–1.39)
Other musculoskeletal problems	30/326 (9.2)	26 (8.8)	4 (12.5)	2.01 (0.65–6.21)
Other				1.21 (0.50–2.96)
Chronic fatigue	3/326 (0.9)	2 (0.7)	1 (3.1)	5.19 (0.37–73.11)
(Free text) High cholesterol	5/326 (1.5)	4 (1.4)	1 (3.1)	2.45 (0.22–27.29)
Chronic pain	15/323 (4.6)	13 (4.4)	2 (6.3)	1.66 (0.34–8.17)
Neck or back problems	29/323 (8.9)	26 (8.8)	3 (9.4)	1.21 (0.33–4.40)
Vision disorders corrected with glasses	158/280 (56.4)	141 (56.2)	17 (56.7)	1.18 (0.54–2.55)
Renal, kidney, bladder, or urogenital system problems	15/326 (4.6)	14 (4.7)	1 (3.1)	0.72 (0.09–5.67)
Chronic lung disease	4/323 (1.2)	4 (1.4)	0 (0)	Unable to calculate
Cancer (any)	21/323 (6.4)	21 (7.1)	0 (0)	Unable to calculate
(Free text) Cleft palate or congenital abnormalities	1/326 (0.3)	1 (0.3)	0 (0)	Unable to calculate
(Free text) Genetic diagnosis	1/326 (0.3)	0 (0)	1 (3.1)	Unable to calculate

*Note*: Odds ratios were calculated using binary logistic regression analysis with probability of class 2 membership as a continuous variable (range: 0–1). We were unable to calculate some odds ratios because of complete separation between classes. (Free text) refers to conditions not listed in the survey but specified by participant(s).

## DISCUSSION

We investigated the phenotypic spectrum of 327 PWS; our model divided the cohort into individuals with common (90%) and complex (10%) presentations. The group with a complex presentation was characterized by higher rates of co‐occurring conditions, particularly neurodevelopmental disorders, lower non‐verbal intelligence, more severe stuttering, and more the negative impact of stuttering.

### Stuttering phenotype

The rates of family history and age at stuttering onset were similar to previous reports.^3^, ^7^ Young adults (15–24 years) showed the most severe stuttering and greatest negative impact of stuttering compared to other ages. There is limited research on stuttering in older adults; however, stuttering severity appears to reduce over time.^5^, ^44^ Positively, we found that negative impact scores also trended lower with age after young adulthood.

### Health and development

One in five participants (*n* = 58, 18%) reported developmental delays, including late talking or walking. Severe or persistent delays were more common in child (40%) than adult (3.8%) participants. This could reflect differences between caregiver‐report and self‐report, with older adults often having minimal information about their developmental history. Recruitment bias may also explain fewer adults with severe or persistent developmental disorders as they were less likely to enrol themselves in research studies compared with caregivers enrolling their children.

Most participants (88%) reported one or more co‐occurring health condition. Consistent with prior research, rates exceeded population prevalence for sleep disturbances,^14^, ^45^ immune system conditions, including allergies and asthma,^15^, ^45^ neurological conditions, including hearing loss and epilepsy,^14^ and anxiety.^6^ Neurodevelopmental disorders were more common in children than adults, with rates of learning difficulties, intellectual disability, autism spectrum disorder, and ADHD similar to previous reports.^13^ Limited data exist for neurodevelopmental disorders in adults who stutter; however, lower adult versus child frequencies aligned with Boyce et al.,^5^ who reported the first 1000 participants from this sample's source population. Moreover, 45 participants (15%) had features of an additional speech disorder, which was between previous estimates based on clinical (16%–46%^46^, ^47^) and community (<7%^48^) samples of PWS.

### Class differences

Individuals in class 2 demonstrated a complex phenotype, with particularly high rates of neurodevelopmental disorders (84% compared with 13% of class 1; OR = 89.73), lower non‐verbal intelligence scores, and included all 25 cases with severe or persistent developmental delay. Class 2 also had more cases with mild or resolved speech disorder, and more health conditions relating to sleep, anxiety, skin, movement, and digestive disorders. Our findings echo a self‐report survey‐based study of 199 adults with a history of childhood stuttering, which did not include formal stuttering assessment. This study identified a larger (72%) and a smaller (28%) group, with the latter showing a more complex presentation, including psychosocial adversities, atopic diseases, and neurodevelopmental disorders; however, frequencies of individual conditions were not reported according to class, limiting comparison with the current study.^15^

The relationship between neurodevelopmental disorders and stuttering may indicate individuals more likely to have a de novo pathogenic gene variant of major effect identified. This is supported by the finding of de novo pathogenic variants in genes associated with neurodevelopmental disorders (*TRIO*, *ZBTB7A*, *PRPF8*, *SPTBN1*) in 4.7% (4 of 85) children who stutter.^11^ Involvement of these genes suggests that stuttering may be a milder manifestation of a neurodevelopmental disorder.

### Strengths and limitations

This study's strengths include the large sample size and comprehensive assessment battery incorporating direct speech assessment, subjective impact measures, and comprehensive health data, enabling investigation of patterns across speech and broader health features.

A key limitation of our study is the skew in age distribution, with only 14% of participants aged 5 to 24 years (*n* = 46), compared to 40% aged 65 and older (*n* = 131). Age distribution will have influenced health condition reporting, with more neurodevelopmental conditions (e.g. autism spectrum disorder) identified in younger participants compared with more age‐related conditions (e.g. arthritis) in older participants. Delayed developmental milestones and diagnoses of developmental delay were reported far less in older participants. The reason for this could be twofold, with older participants demonstrating recall bias (i.e. no knowledge of, or access to, information about early development) and generational differences in diagnosis. Importantly, a skew in age distribution may have influenced latent class assignment, with class 2 being a mean 24 years younger and also more frequently reporting neurodevelopmental conditions more common in younger participants. Future research should consider using standardized assessments (e.g. language assessment) to address self‐report limitations in participants of different ages. Additionally, while videoconference is reliable for stuttering assessment^25^, ^26^, ^27^ and perceptual speech assessment,^27^, ^31^, ^32^, ^33^, ^34^, ^35^ it is possible that some subtle speech features could have been missed, which may have otherwise been observed during in‐person speech assessment.

## Conclusions

This study provides evidence for common and complex stuttering phenotypes. The complex group had more frequent co‐occurring health conditions, particularly neurodevelopmental disorders and anxiety, lower non‐verbal intelligence, more severe stuttering, and a more negative impact of stuttering. Findings highlight the heterogeneous nature of stuttering and the need for multidisciplinary support to address all features associated with this disorder.

## Supporting information


**Appendix S1:** Core health survey, adult version (genome‐wide association study).

**Appendix S2:** Health and development survey (present study).

**Appendix S3:** Speech assessment protocol.

**Figure S1:** Participant recruitment and selection of final analytic sample.

**Figure S2:** Percentage of participants demonstrating stuttering behaviours.

**Figure S3:** Summary of participant‐reported health conditions compared with population prevalence rates, by age.

**Table S1:** Indicator variables and response categories used in latent class analysis.

**Table S2:** Demographic variables by participation status for current study.

**Table S3:** Covert strategies used to control or avoid stuttering during conversation sample.

**Table S4:** Speech assessment data and participant‐reported speech diagnoses.

**Table S5:** Survey questions about development in early life.

**Table S6:** Spearman's rank correlations between indicator variables.

**Table S7:** Indicator variable response categories, stratified by latent class (sensitivity test 1).

**Table S8:** Indicator variable response categories, stratified by latent class (sensitivity test 2).

**Table S9:** Indicator variable response categories, stratified by latent class (sensitivity test 3).

**Table S10:** Indicator variable response categories, stratified by latent class (sensitivity test 4).

**Table S11:** Stuttering behaviours, stratified by latent class.

## Data Availability

The data that support the findings of this study are available from the corresponding author upon reasonable request. The data are not publicly available due to privacy restrictions.
