# Supplementary material for: Developmental stuttering with common and complex phenotypes
Source: Dev Med Child Neurol. 2026 Feb 23;68(10):1460–75. doi: 10.1111/dmcn.70208 (PMC13545487; doi:10.1111/dmcn.70208)
Supplement: Supplementary file 1 [file DMCN-68-1460-s001.docx]

**Table S6.**

*Spearman’s rank correlations between indicator variables.*

|  | Family history of stuttering  *r_s_* (95% CI) | Age of stuttering onset  *r_s_* (95% CI) | Stuttering presence and severity  *r_s_* (95% CI) | Development in early life  *r_s_* (95% CI) | Co-occurring speech disorder  *r_s_* (95% CI) | Nonverbal intelligence  *r_s_* (95% CI) |
| --- | --- | --- | --- | --- | --- | --- |
| Age of stuttering onset | -0.08 (-0.19, 0.03) |  |  |  |  |  |
| Stuttering presence and severity | -0.05 (-0.16, 0.07) | 0.02 (-0.10, 0.13) |  |  |  |  |
| Development in early life | -0.01 (-0.12, 0.10) | 0.01 (-0.11, 0.11) | 0.13 (0.02, 0.24) |  |  |  |
| Co-occurring speech disorder | 0.01 (-0.11, 0.12) | -0.02 (-0.13, 0.10) | 0.16 (0.05, 0.27) | 0.20 (0.09, 0.31) |  |  |
| Nonverbal intelligence | 0.08 (-0.04, 0.20) | 0.02 (-0.10, 0.14) | 0.04 (-0.08, 0.16) | 0.17 (0.05, 0.28) | 0.10 (-0.02, 0.22) |  |
| Co-occurring health conditions | -0.04 (-0.15, 0.07) | 0.04 (-0.07, 0.15) | 0.02 (-0.10, 0.13) | 0.10 (-0.01, 0.20) | 0.03 (-0.08, 0.15) | 0.03 (-0.09, 0.15) |

*Note*. *n* = 293–324
