# Supplementary material for: Developmental stuttering with common and complex phenotypes
Source: Dev Med Child Neurol. 2026 Feb 23;68(10):1460–75. doi: 10.1111/dmcn.70208 (PMC13545487; doi:10.1111/dmcn.70208)
Supplement: Supplementary file 2 [file DMCN-68-1460-s002.docx]

**Table S7.**

*Frequency counts and odds ratios for indicator variable response categories, stratified by latent class (sensitivity test 1: participants with resolved stuttering excluded, total n = 313).*

|  | All  *n* = 313 | Class 1  *n* = 283 | Class 2  *n* = 30 |  |  |
| --- | --- | --- | --- | --- | --- |
| **Age at recruitment (years)** |  |  |  |  |  |
| Mean (SD) | 52.6 (22.1) | 55.2 (20.7) | 28.8 (21.3) |  |  |
| Range | 5–89 | 5–89 | 8–77 |  |  |
| **Indicator variable & response category** | *n*/313 (%) | *n*/283 (%) | *n*/30 (%) | Odds ratio (OR)  [95% CI] | Interpretation of odds ratio:  Class 2 vs Class 1 |
| 1. Family history of stuttering |  |  |  |  |  |
| a: No family history | 114/311 (37) | 102/281 (36) | 12/30 (40) |  |  |
| b: Positive family history | 197/311 (63) | 179/281 (64) | 18/30 (60) | 0.83 [0.37, 1.86] | Lower odds of positive family history |
| 2. Age of stuttering onset |  |  |  |  |  |
| a: Onset ≤4 years | 176/308 (57) | 154/278 (55) | 23/30 (73) | 2.39 [0.99, 5.75] | Higher odds of onset ≤4 years |
| b: Onset 5–8 years | 103/308 (33) | 99/278 (36) | 4/30 (13) | 0.14 [0.04, 0.52] | Lower odds of onset 5–8 years |
| c: Onset ≥9 years | 29/308 (9) | 25/278 (9) | 4/30 (13) | 2.67, [0.92, 7.76] | Higher odds of onset ≥9 years |
| 3. Stuttering presence and severity |  |  |  |  |  |
| a: Mild | 205/282 (73) | 192/256 (75) | 13/26 (50) | 0.27 [0.11, 0.63] | Lower odds of mild stuttering |
| b: Moderate-to-severe | 77/282 (27) | 64/256 (25) | 13/26 (50) | 3.76 [1.59, 8.88] | Higher odds of moderate-to-severe stuttering |
| 4. Development in early life |  |  |  |  |  |
| a: Typical development | 255/311 (82) | 255/281 (91) | 0/30 (0) | Unable to calculate | All cases classified to Class 1 |
| b: Mild or resolved developmental delay | 32/311 (10) | 26/281 (9) | 6/30 (20) | 6.26 [2.46, 15.96] | Higher odds of mild or resolved delay |
| c: Severe or persistent delay/disorder | 24/311 (8) | 0/281 (0) | 24/30 (80) | Unable to calculate | All cases classified to Class 2 |
| 5. Co-occurring speech disorder |  |  |  |  |  |
| a: No other speech disorder | 238/283 (84) | 225/256 (88) | 13/27 (48) | 0.09 [0.04, 0.23] | Lower odds of no other speech disorder |
| b: Mild or resolved speech disorder | 33/283 (12) | 21/256 (8) | 12/27 (45) | 10.75 [4.17, 27.74] | Higher odds of mild or resolved speech disorder |
| c: Moderate-to-severe or persistent speech disorder | 12/283 (4) | 10/256 (4) | 2/27 (7) | 3.42 [0.77, 15.19] | Higher odds of severe or persistent speech disorder |
| 6. Nonverbal intelligence |  |  |  |  |  |
| a: Average or above average score | 218/252 (87) | 201/227 (89) | 17/25 (68) |  |  |
| b: Below average score | 34/252 (13) | 26/227 (11) | 8/25 (32) | 4.04 [1.50, 10.88] | Higher odds of below average score |
| 7. Co-occurring health conditions |  |  |  |  |  |
| a: No other diagnoses | 39/312 (13) | 38/282 (14) | 1/30 (3) | 0.04 [<0.01, 1.42] | Lower odds of no other diagnoses |
| b: Diagnoses in 1–3 health domains | 173/312 (55) | 162/282 (57) | 11/30 (37) | 0.42 [0.19, 0.95] | Lower odds of diagnoses in 1–3 health domains |
| c: Diagnoses in ≥4 health domains | 100/312 (32) | 82/282 (29) | 18/30 (60) | 4.62 [2.04, 10.48] | Higher odds of diagnoses in ≥4 health domains |
| **Total** | 313 | 283 | 30 |  |  |

*Note.* OR calculated using binary logistic regression analysis with probability of Class 2 membership as a continuous variable (ranging 0–1). Unable to calculate some ORs due to complete separation of data between classes. Sample sizes for individual measures ranged from *n* = 252 to 312.
