# Supplementary material for: Developmental stuttering with common and complex phenotypes
Source: Dev Med Child Neurol. 2026 Feb 23;68(10):1460–75. doi: 10.1111/dmcn.70208 (PMC13545487; doi:10.1111/dmcn.70208)
Supplement: Supplementary file 3 [file DMCN-68-1460-s003.docx]

**Figure S3.**

*Frequency of participant-reported health conditions compared with population prevalence rates, by age. a) sleep domain, b) conditions in neurological domain, c) conditions in immune system domain, d) conditions in mental health domain, e) conditions in musculoskeletal domain, f) conditions in cardiovascular domain, g) conditions in neurodevelopment domain, h) conditions in gut and digestive system domain, i) conditions in endocrine system domain, j) other conditions.*

*a) Sleep domain*

*b) Conditions in neurological domain*

*c) Conditions in immune system domain*

*d) Conditions in mental health domain*

*e) Conditions in musculoskeletal domain*

*f) Conditions in cardiovascular domain*

*g) Conditions in neurodevelopment domain*

*h) Conditions in gut and digestive system domain*

*i) Conditions in endocrine system domain*

*j) Other conditions*

*Note.* Population prevalence estimates based on Australian Bureau of Statistics data, where available, with total population prevalence estimate reported where age-group estimates could not be found.
