# Supplementary material for: Developmental stuttering with common and complex phenotypes
Source: Dev Med Child Neurol. 2026 Feb 23;68(10):1460–75. doi: 10.1111/dmcn.70208 (PMC13545487; doi:10.1111/dmcn.70208)
Supplement: Supplementary file 5 [file DMCN-68-1460-s005.docx]

**Table S9.**

*Frequency counts and odds ratios for indicator variable response categories, stratified by latent class (sensitivity test 3: youngest group of participants <15 years excluded, total n = 295).*

|  | All  *n* = 295 | Class 1  *n* = 270 | Class 2  *n* = 25 |  |  |
| --- | --- | --- | --- | --- | --- |
| **Age at recruitment (years)** |  |  |  |  |  |
| Mean (SD) | 57.0 (18.0) | 58.1 (17.5) | 44.4 (18.5) |  |  |
| Range | 15–89 | 15–89 | 15–77 |  |  |
| **Indicator variable & response category** | *n*/295 (%) | *n*/270 (%) | *n*/25 (%) | Odds ratio (OR)  [95% CI] | Interpretation of odds ratio:  Class 2 vs Class 1 |
| 1. Family history of stuttering |  |  |  |  |  |
| a: No family history | 106/294 (36) | 98/269 (36) | 8/25 (32) |  |  |
| b: Positive family history | 188/294 (64) | 171/269 (64) | 17/25 (68) | 1.11 [0.42, 2.97] | Higher odds of positive family history |
| 2. Age of stuttering onset |  |  |  |  |  |
| a: Onset ≤4 years | 160/292 (55) | 147/267 (55) | 13/25 (52) | 1.16 [0.45, 2.97] | Higher odds of onset ≤4 years |
| b: Onset 5–8 years | 103/292 (35) | 101/267 (38) | 2/25 (8) | 0.02 [<0.01, 0.31] | Lower odds of onset 5–8 years |
| c: Onset ≥9 years | 29/292 (10) | 19/267 (7) | 10/76 (40) | 13.16, [4.51, 38.42] | Higher odds of onset ≥9 years |
| 3. Stuttering presence and severity |  |  |  |  |  |
| a: Resolved | 13/267 (5) | 12/245 (5) | 1/22 (5) | 0.79 [0.06, 9.78] | Lower odds of resolved stuttering |
| b: Mild | 184/267 (69) | 172/245 (70) | 12/22 (55) | 0.43 [0.16, 1.19] | Lower odds of mild stuttering |
| c: Moderate-to-severe | 70/267 (26) | 61/245 (25) | 9/22 (41) | 2.56 [0.91, 7.19] | Higher odds of moderate-to-severe stuttering |
| 4. Development in early life |  |  |  |  |  |
| a: Typical development | 252/295 (85) | 249/270 (92) | 3/25 (12) | <0.01 [<0.01, 0.01] | Lower odds of typical development |
| b: Mild or resolved developmental delay | 30/295 (10) | 21/270 (8) | 9/25 (36) | 10.73 [3.70, 31.14] | Higher odds of mild or resolved delay |
| c: Severe or persistent delay/disorder | 13/295 (4) | 0/270 (0) | 13/25 (52) | Unable to calculate | All cases classified to Class 2 |
| 5. Co-occurring speech disorder |  |  |  |  |  |
| a: No other speech disorder | 231/267 (87) | 221/244 (91) | 10/23 (43) | 0.04 [0.01, 0.13] | Lower odds of no other speech disorder |
| b: Mild or resolved speech disorder | 25/267 (9) | 15/244 (6) | 10/23 (43) | 17.60 [5.58, 55.49] | Higher odds of mild or resolved speech disorder |
| c: Moderate-to-severe or persistent speech disorder | 11/267 (4) | 8/244 (3) | 3/23 (13) | 7.80 [1.67, 36.42] | Higher odds of severe or persistent speech disorder |
| 6. Nonverbal intelligence |  |  |  |  |  |
| a: Average or above average score | 204/236 (86) | 189/216 (88) | 15/20 (75) |  |  |
| b: Below average score | 32/236 (14) | 27/216 (12) | 5/20 (25) | 3.62 [1.08, 12.16] | Higher odds of below average score |
| 7. Co-occurring health conditions |  |  |  |  |  |
| a: No other diagnoses | 33/295 (11) | 31/270 (11) | 2/25 (8) | 0.44 [0.07, 2.96] | Lower odds of no other diagnoses |
| b: Diagnoses in 1–3 health domains | 164/295 (56) | 160/270 (59) | 4/25 (16) | 0.04 [0.01, 0.20] | Lower odds of diagnoses in 1–3 health domains |
| c: Diagnoses in ≥4 health domains | 98/295 (33) | 79/270 (29) | 19/25 (76) | 23.79 [6.30, 89.78] | Higher odds of diagnoses in ≥4 health domains |
| **Total** | 295 | 270 | 25 |  |  |

*Note.* OR calculated using binary logistic regression analysis with probability of Class 2 membership as a continuous variable (ranging 0–1). Unable to calculate some ORs due to complete separation of data between classes. Sample sizes for individual measures ranged from *n* = 236–295.
