# Supplementary material for: Developmental stuttering with common and complex phenotypes
Source: Dev Med Child Neurol. 2026 Feb 23;68(10):1460–75. doi: 10.1111/dmcn.70208 (PMC13545487; doi:10.1111/dmcn.70208)
Supplement: Supplementary file 6 [file DMCN-68-1460-s006.docx]

**Table S4.**

*Speech assessment data and participant-reported speech diagnoses.*

| Characteristics of co-occurring speech disorders | | *n*/total | % |  |
| --- | --- | --- | --- | --- |
| Previous diagnosis | | | | |
|  | Articulation or phonological disorder | 15/323 | 4.6 |  |
|  | Apraxia of speech | 1/323 | 0.3 |  |
|  | Cluttering | 1/323 | 0.3 |  |
|  | [Free text] Mixing up sounds | 10/323 | 3.1 |  |
| Oral motor examination | |  |  |  |
| Structural assessment – atypical features noted: | |  |  |  |
|  | Teeth e.g., additional, not in alignment | 13/296 | 4.4 |  |
|  | Lips e.g., asymmetry | 5/296 | 1.7 |  |
|  | Tongue e.g., enlarged | 4/296 | 1.4 |  |
|  | Mandible e.g., malocclusion | 2/296 | 0.7 |  |
|  | Velopharynx/palate e.g., cleft palate | 1/296 | 0.3 |  |
| Functional assessment – atypical features noted during: | |  |  |  |
|  | Polysyllabic alternation (repeat pataka) | 40/296 | 13.5 |  |
|  | Sequencing (blow, lick lips) | 23/296 | 7.8 |  |
|  | Oral nasal alteration (repeat u-n-a) | 10/296 | 3.4 |  |
|  | Tongue elevation | 9/296 | 3.0 |  |
|  | Alternating tongue protrusion | 8/296 | 2.7 |  |
|  | Monosyllabic alternation (repeat papapa) | 7/296 | 2.4 |  |
|  | Tongue protrusion (isolated) | 7/296 | 2.4 |  |
|  | Lip seal (puff cheeks) | 7/296 | 2.4 |  |
|  | Alternating lip protrusion/retraction | 6/296 | 2.0 |  |
|  | Bite lower lip (/f/) | 5/296 | 1.7 |  |
|  | Tongue interdental | 5/296 | 1.7 |  |
|  | Lip protrusion/retraction (isolated) | 4/296 | 1.4 |  |
|  | Velopharynx and palate e.g., reduced movement | 3/296 | 1.0 |  |
| Perceptual speech assessment* | |  |  |  |
| Speech sound errors | |  |  |  |
|  | Articulation errors or phonological errors | 22/296 | 7.4 |  |
| Prosodic errors | |  |  |  |
|  | Altered rate e.g., fast or slow | 18/296 | 6.1 |  |
|  | Stress errors e.g., excess and equal stress | 5/296 | 1.7 |  |
|  | Syllable segregation/ segmentation | 4/296 | 1.4 |  |
| Lengthened & disrupted coarticulatory transitions | |  |  |  |
|  | Transposition errors | 9/296 | 3.0 |  |
|  | Articulatory groping | 7/296 | 2.4 |  |
|  | Difficulty maintaining syllable integrity | 4/296 | 1.4 |  |
|  | Substitution, distortion, or addition errors | 3/296 | 1.0 |  |
|  | Epenthesis (intrusive schwa) | 1/296 | 0.3 |  |
| Voice/ respiration | |  |  |  |
|  | Pitch variability | 18/296 | 6.1 |  |
|  | Volume/loudness control | 7/296 | 2.4 |  |
|  | Inappropriate voice quality e.g., breathy, breaks in phonation | 5/296 | 1.7 |  |
|  | Altered resonance e.g., hypernasality | 3/296 | 1.0 |  |
|  | Altered respiration e.g., forced inspiration-expiration | 1/296 | 0.3 |  |
| Total | |  |  |  |
|  | No co-occurring speech disorder | 251/296 | 84.8 |  |
|  | Mild or resolved speech disorder | 33/296 | 11.1 |  |
|  | Severe or persistent speech disorder | 12/296 | 4.1 |  |

*Note.* * Rated during motor speech battery and conversation.
