# Supplementary material for: Developmental stuttering with common and complex phenotypes
Source: Dev Med Child Neurol. 2026 Feb 23;68(10):1460–75. doi: 10.1111/dmcn.70208 (PMC13545487; doi:10.1111/dmcn.70208)
Supplement: Supplementary file 7 [file DMCN-68-1460-s007.docx]

**Table S10.**

*Frequency counts and odds ratios for indicator variable response categories, stratified by latent class (sensitivity test 4: all participants with age as a covariate, total n = 327).*

|  | All  *n =* 327 | Class 1  *n =* 250 | Class 2  *n* = 45 | Class 3  *n* = 32 |
| --- | --- | --- | --- | --- |
| **Age at recruitment (years)** |  |  |  |  |
| Mean (SD) | 52.3 (22.1) | 61.6 (14.8) | 22.7 (12.6) | 21.5 (14.0) |
| Range | 5–89 | 15–89 | 5–44 | 8–56 |
| **Indicator variable & response category** | *n*/327 (%) | *n*/250 (%) | *n*/45 (%) | *n*/32 (%) |
| 1. Family history of stuttering |  |  |  |  |
| a: No family history | 118/324 (36) | 93/248 (37) | 9/44 (20) | 16/32 (50) |
| b: Positive family history | 207/324 (64) | 156/248 (63) | 35/44 (80) | 16/32 (50) |
| 2. Age of stuttering onset |  |  |  |  |
| a: Onset ≤4 years | 187/322 (58) | 122/247 (49) | 41/43 (95) | 24/32 (75) |
| b: Onset 5–8 years | 106/322 (33) | 100/247 (40) | 2/43 (5) | 4/32 (13) |
| c: Onset ≥9 years | 29/322 (9) | 25/247 (10) | 0/43 (0) | 4/32 (13) |
| 3. Stuttering presence and severity |  |  |  |  |
| a: Resolved | 14/296 (5) | 7/229 (3) | 6/40 (15) | 1/27 (4) |
| b: Mild | 205/296 (69) | 157/229 (69) | 34/40 (85) | 14/27 (52) |
| c: Moderate-to-severe | 77/296 (26) | 65/229 (28) | 0/40 (0) | 12/27 (44) |
| 4. Development in early life |  |  |  |  |
| a: Typical development | 267/325 (82) | 224/250 (90) | 43/44 (98) | 0/31 (0) |
| b: Mild or resolved developmental delay | 33/325 (10) | 26/250 (10) | 1/44 (2) | 6/31 (19) |
| c: Severe or persistent delay/disorder | 25/325 (8) | 0/250 (0) | 0/44 (0) | 25/31 (81) |
| 5. Co-occurring speech disorder |  |  |  |  |
| a: No other speech disorder | 252/297 (85) | 198/228 (87) | 35/40 (88) | 19/29 (66) |
| b: Mild or resolved speech disorder | 33/297 (11) | 20/228 (9) | 5/40 (13) | 8/29 (28) |
| c: Moderate-to-severe or persistent speech disorder | 12/297 (4) | 10/228 (4) | 0/40 (0) | 1/29 (7) |
| 6. Nonverbal intelligence |  |  |  |  |
| a: Average or above average score | 226/264 (86) | 177/201 (88) | 31/37 (84) | 18/26 (69) |
| b: Below average score | 38/264 (14) | 24/201 (12) | 6/37 (16) | 8/26 (31) |
| 7. Co-occurring health conditions |  |  |  |  |
| a: No other diagnoses | 39/326 (12) | 19/250 (8) | 19/44 (43) | 1/32 (3) |
| b: Diagnoses in 1–3 health domains | 183/326 (56) | 148/250 (59) | 21/44 (48) | 14/32 (44) |
| c: Diagnoses in ≥4 health domains | 104/326 (32) | 83/250 (33) | 4/44 (9) | 17/32 (53) |
| **Total** | 327 | 250 | 45 | 32 |

*Note.* Sample sizes for individual measures ranged from *n* = 264 to 326.
