# Supplementary material for: Developmental stuttering with common and complex phenotypes
Source: Dev Med Child Neurol. 2026 Feb 23;68(10):1460–75. doi: 10.1111/dmcn.70208 (PMC13545487; doi:10.1111/dmcn.70208)
Supplement: Supplementary file 8 [file DMCN-68-1460-s008.docx]

**Appendix S3.**

*Speech assessment protocol*

| Assessment measure | Method of collection | Contribution to statistical analyses | Relation to phenotype |
| --- | --- | --- | --- |
| Stuttering characteristics during conversation sample | | | |
| Severity rating | Rated by clinician and participant or caregiver using 10-point scale. | Demographics, Indicator variable 3 | Clinical measure of severity |
| Primary stuttering behaviours | Rated by clinician: blocks, prolongations, sound repetitions, part-word repetitions, word repetitions, phrase repetitions, and other. | Stuttering phenotype | Behavioural manifestation |
| Secondary stuttering behaviours | Rated by clinician: head movements, rapid eye blinking, facial grimacing, making distracting sounds, trunk or limb movements, freezing or becoming rigid, sweating, and other. | Stuttering phenotype | Associated behaviours |
| Covert behaviours | Reported by participant or caregiver: avoiding certain words or sounds, using strategies learned in speech therapy, changing the order of words in sentences, starting to speak then giving up, and other. | Stuttering phenotype | Attempts to avoid, cope, manage |
| Family history of stuttering | | | |
| Pedigree interview | Reported by participant or caregiver, recorded by clinician using standardised pedigree nomenclature (29). | Indicator variable 1 | Family history |
| Features of co-occurring speech disorders* during oral motor and speaking tasks^ | | | |
| Common speech disorders (articulation, phonological disorder) | Rated perceptually by clinician based on oral motor and speaking tasks and compared against clinical norms (36). If features present, classified as developmentally appropriate, delayed, or atypical. | Participant demographics, Indicator variable 5 | Features of co-occurring speech disorders |
| Uncommon speech disorders (dysarthria, apraxia of speech) | Rated perceptually by clinician based on oral motor and speaking tasks using Mayo Clinic Dysarthria Rating Scale (37), perceptually salient characteristics of apraxia of speech (37), and Childhood Apraxia of Speech Checklist (38), focusing on three key criteria: inconsistent speech production, disrupted and prolonged co-articulatory transitions, and prosodic errors. If features present, classified as age-appropriate, mild, or moderate-to-severe. | Participant demographics, Indicator variable 5 | Features of co-occurring speech disorders |

*Note.* 10-point stuttering severity rating scale: 1 = *no stuttering,* 10 = *extremely severe stuttering*. *Co-occurring speech disorders were classified as developmentally common (articulation disorders and phonological disorders) or developmentally uncommon (motor speech disorders, including dysarthria and apraxia of speech). Articulation errors result from incorrect placement of articulators (e.g., tongue, lips) or incorrect manner of production (e.g., voiced, voiceless) for sounds. Common articulation errors include interdental lisps, where the /s/ sound is produced anteriorly. Phonological errors result from poor understanding of the use of sound patterns in a language. Errors may involve patterns of substitutions or omissions of sounds, for example, gliding errors where “wabbit” is produced for “rabbit,” or cluster reduction where “pray” is produced for “spray.” Dysarthria results from problems with range, rate, strength, and co-ordination of the muscles used for speech, and may affect domains of articulation, resonance, respiration, phonation. Apraxia of speech results from difficulty planning and sequencing motor movements for speech. ^Oral motor and speaking tasks included the Oral and Speech Motor Control Protocol (30) and a motor speech battery, including automatic speech (naming the days of the week), a reading passage, picture a description task, a 2-minute monologue, and a conversation sample.
