# Supplementary material for: Developmental stuttering with common and complex phenotypes
Source: Dev Med Child Neurol. 2026 Feb 23;68(10):1460–75. doi: 10.1111/dmcn.70208 (PMC13545487; doi:10.1111/dmcn.70208)
Supplement: Supplementary file 9 [file DMCN-68-1460-s009.docx]

**Table S3.**

*Covert strategies used to control or avoid stuttering during conversation sample.*

| Strategy | *n*/282 | % |
| --- | --- | --- |
| Avoiding certain words or sounds | 47 | 16.7 |
| Using strategies learned in speech therapy (e.g., smooth speech) | 31 | 11.0 |
| Changing word order in sentences | 30 | 10.6 |
| Starting to speak then giving up | 8 | 2.8 |
| Other techniques | 59 | 20.9 |
| Slowing down | 11 | 3.9 |
| Breathing techniques | 7 | 2.5 |
| Pausing | 4 | 1.4 |
| Extending or stretching sounds | 4 | 1.4 |
| Thinking or planning before speaking | 3 | 1.0 |
| Timing or foot tapping | 2 | 0.7 |
| Acting or using a different voice | 2 | 0.7 |
| Other (unspecified) | 26 | 9.2 |
