# Supplementary material for: Developmental stuttering with common and complex phenotypes
Source: Dev Med Child Neurol. 2026 Feb 23;68(10):1460–75. doi: 10.1111/dmcn.70208 (PMC13545487; doi:10.1111/dmcn.70208)
Supplement: Supplementary file 10 [file DMCN-68-1460-s010.docx]

**Figure S2.**

*Percentage of participants demonstrating stuttering behaviours in n=282 participants with persistent stuttering, stratified by age. a) Primary stuttering behaviours, b) Secondary stuttering behaviours.*

 *Note.* For primary stuttering behaviours, *other* = excessive insertion of “um,” “uh” or “you know” (*n* = 8), atypical mid-word block (*n* = 2), insertion of schwa (unstressed syllable; *n* = 1). For secondary stuttering behaviours, *other* = eye roll (*n* = 4), tongue click (*n* = 3), sharp inhalation (*n* = 2), tongue thrust (*n* = 1), gulp/swallow (*n* = 1).
