# Supplementary material for: Developmental stuttering with common and complex phenotypes
Source: Dev Med Child Neurol. 2026 Feb 23;68(10):1460–75. doi: 10.1111/dmcn.70208 (PMC13545487; doi:10.1111/dmcn.70208)
Supplement: Supplementary file 11 [file DMCN-68-1460-s011.docx]

**Table S8.**

*Frequency counts and odds ratios for indicator variable response categories, stratified by latent class (sensitivity test 2: only complete cases retained, total n = 260).*

|  | All  *n* = 260 | Class 1  *n* = 234 | Class 2  *n* = 26 |  |  |
| --- | --- | --- | --- | --- | --- |
| **Age at recruitment (years)** |  |  |  |  |  |
| Mean (SD) | 52.2 (22.0) | 54.6 (20.7) | 29.9 (21.5) |  |  |
| Range | 6–85 | 6–85 | 11–77 |  |  |
| **Indicator variable & response category** | *n*/260 (%) | *n*/234 (%) | *n*/26 (%) | Odds ratio (OR)  [95% CI] | Interpretation of odds ratio: Class 2 vs Class 1 |
| 1. Family history of stuttering |  |  |  |  |  |
| a: No family history | 93 (36) | 82 (35) | 11 (42) |  |  |
| b: Positive family history | 167 (64) | 152 (65) | 15 (58) | 0.70 [0.29, 1.70] | Lower odds of positive family history |
| 2. Age of stuttering onset |  |  |  |  |  |
| a: Onset ≤4 years | 156 (60) | 137 (59) | 19 (73) | 3.30 [1.14, 9.53] | Higher odds of onset ≤4 years |
| b: Onset 5–8 years | 80 (31) | 77 (33) | 3 (12) | 0.07 [0.01, 0.43] | Lower odds of onset 5–8 years |
| c: Onset ≥9 years | 24 (9) | 20 (9) | 4 (15) | 2.43, [0.70, 8.39] | Higher odds of onset ≥9 years |
| 3. Stuttering presence and severity |  |  |  |  |  |
| a: Resolved | 12 (5) | 11 (5) | 1 (4) | 0.52 [0.04, 6.80] | Lower odds of resolved stuttering |
| b: Mild | 182 (70) | 168 (72) | 14 (54) | 0.35 [0.14, 0.87] | Lower odds of mild stuttering |
| c: Moderate-to-severe | 66 (25) | 55 (23) | 11 (42) | 3.35 [1.35, 8.32] | Higher odds of moderate-to-severe stuttering |
| 4. Development in early life |  |  |  |  |  |
| a: Typical development | 211 (81) | 209 (89) | 2 (8) | <0.01 [<0.01, 0.01] | Lower odds of typical development |
| b: Mild or resolved developmental delay | 28 (11) | 25 (11) | 3 (11) | 1.61 [0.46, 5.72] | Higher odds of mild delay |
| c: Severe or persistent delay/disorder | 21 (8) | 0 (0) | 21 (81) | Unable to calculate | All cases classified to Class 2 |
| 5. Co-occurring speech disorder |  |  |  |  |  |
| a: No other speech disorder | 220 (85) | 207 (88) | 13 (50) | 0.07 [0.02, 0.18] | Lower odds of no other speech disorder |
| b: Mild or resolved speech disorder | 31 (12) | 21 (9) | 10 (38) | 10.56 [3.83, 29.08] | Higher odds of mild or resolved speech disorder |
| c: Moderate-to-severe or persistent speech disorder | 9 (3) | 6 (3) | 3 (12) | 9.00 [1.89, 42.80] | Higher odds of severe or persistent speech disorder |
| 6. Nonverbal intelligence |  |  |  |  |  |
| a: Average or above average score | 223 (86) | 204 (87) | 19 (73) |  |  |
| b: Below average score | 37 (14) | 30 (13) | 7 (27) | 2.68 [0.94, 7.65] | Higher odds of below average score |
| 7. Co-occurring health conditions |  |  |  |  |  |
| a: No other diagnoses | 30 (12) | 30 (13) | 0 (0) | Unable to calculate | All cases classified to Class 1 |
| b: Diagnoses in 1–3 health domains | 148 (57) | 140 (60) | 8 (31) | 0.14 [0.05, 0.39] | Lower odds of diagnoses in 1–3 health domains |
| c: Diagnoses in ≥4 health domains | 82 (32) | 64 (27) | 18 (69) | 28.51 [8.12, 100.16] | Higher odds of diagnoses in ≥4 health domains |
| **Total** | 260 | 234 (90) | 26 (10) |  |  |

*Note.* OR calculated using binary logistic regression analysis with probability of Class 2 membership as a continuous variable (ranging 0–1). Unable to calculate some ORs due to complete separation of data between classes.
