# Supplementary material for: Developmental stuttering with common and complex phenotypes
Source: Dev Med Child Neurol. 2026 Feb 23;68(10):1460–75. doi: 10.1111/dmcn.70208 (PMC13545487; doi:10.1111/dmcn.70208)
Supplement: Supplementary file 12 [file DMCN-68-1460-s012.docx]

**Table S11.**

*Frequency counts and odds ratios for stuttering behaviours, stratified by latent class.*

| Stuttering behaviour | Total *n* = 282 | | Class 1 *n* = 255 | | Class 2 *n* = 27 | | Odds ratio (OR)  [95% CI] |
| --- | --- | --- | --- | --- | --- | --- | --- |
|  | *n* | % | *n* | *%* | *n* | *%* |  |
| Primary stuttering behaviour | |  |  |  |  |  |  |
| Block | 177 | 62.3 | 161 | 63.1 | 16 | 59.3 | 1.12 [0.47, 2.71] |
| Prolongation | 87 | 30.9 | 75 | 29.4 | 12 | 44.4 | 1.91 [0.80, 4.52] |
| Sound repetition | 214 | 75.9 | 191 | 74.9 | 23 | 85.2 | 2.18 [0.69, 6.90] |
| Part-word repetition | 119 | 42.2 | 108 | 42.4 | 11 | 40.7 | 0.85 [0.36, 2.02] |
| Word repetition | 121 | 42.9 | 111 | 43.5 | 10 | 37.0 | 0.70 [0.29, 1.67] |
| Phrase repetition | 76 | 27.0 | 72 | 28.2 | 4 | 14.8 | 0.37 [0.11, 1.21] |
| Other | 17 | 6.0 | 13 | 5.1 | 4 | 14.8 | 2.43 [0.60, 9.85] |
| Secondary stuttering behaviour | |  |  |  |  |  |  |
| Facial grimacing | 52 | 18.4 | 39 | 15.3 | 13 | 48.2 | 6.61 [2.69, 16.25] |
| Rapid eye-blinking | 45 | 16.0 | 35 | 13.7 | 10 | 37.0 | 4.37 [1.73, 11.04] |
| Head movements | 33 | 11.7 | 25 | 9.8 | 8 | 29.6 | 5.47 [2.05, 14.60] |
| Making distracting sounds | 21 | 7.4 | 17 | 6.7 | 4 | 14.8 | 2.68 [0.76, 9.44] |
| Trunk or limb movements | 11 | 3.9 | 7 | 2.8 | 4 | 14.8 | 8.12 [2.00, 33.01] |
| Freezing or becoming rigid | 3 | 1.1 | 1 | 0.4 | 2 | 7.4 | 27.18 [1.93, 381.75] |
| Sweating | 2 | 0.7 | 1 | 0.4 | 1 | 3.7 | 11.67 [0.56, 244.11] |
| Other | 33 | 11.7 | 27 | 10.6 | 6 | 22.2 | 3.00 [1.06, 8.55] |

*Note*. OR calculated using binary logistic regression analysis with probability of Class 2 membership as a continuous variable (ranging 0–1).
