# Supplementary material for: Developmental stuttering with common and complex phenotypes
Source: Dev Med Child Neurol. 2026 Feb 23;68(10):1460–75. doi: 10.1111/dmcn.70208 (PMC13545487; doi:10.1111/dmcn.70208)
Supplement: Supplementary file 13 [file DMCN-68-1460-s013.docx]

**Appendix S2.**

*Health and development survey (present study).*

| Survey question | Response options | Contribution to statistical analyses |
| --- | --- | --- |
| Demographic information | |  |
| *Do you speak any languages other than English?* | |  |
|  | - No - Yes | Demographic summary |
| If “yes,” *What language do you prefer to speak?* | |  |
|  | Free text | Demographic summary |
| Communication and motor development | |  |
| *When did you say your first word?* | |  |
|  | - < 12 months - 12-15 months - 15-18 months - >18 months - I don't know | Indicator variable 4 |
| *When did you begin talking in short sentences?* | |  |
|  | - 2-3 years - 4-5 years, - 6-7 years, - >8 years, - I don't know | Indicator variable 4 |
| *Were your parents/guardians ever concerned that you may have lost language for 3 months or more after you had mastered these skills?* | | |
|  | - Don’t know - No - Yes | Indicator variable 4 |
| *Were your parents/guardians ever concerned that you may have lost other skills (e.g. fine or gross motor control, self-help skills, imaginative or creative play, or social responsiveness) for 3 months or more, after you had mastered these skills?* | | |
|  | - Don’t know - No - Yes | Indicator variable 4 |
| *When did you begin walking?* | |  |
|  | - 9-12 months - 13-15 months - >16 months - I don't know | Indicator variable 4 |
| Medical history |  |  |
| *Has your hearing been tested?* | |  |
|  | - Don’t know - No - Yes | Indicator variable 7 |
| If “yes,” *Is your hearing normal or impaired?* | |  |
|  | - Normal - Impaired - I don't know | Indicator variable 7 |
| If “impaired,” *Please describe hearing impairment (type and degree of hearing impairment e.g., mild conductive hearing loss)* | | |
|  | Free text | Indicator variable 7 |
| *Do you have a history of sleep disturbance? (e.g., slow to fall asleep, frequent waking, early waking, sleep apnoea)* | | |
|  | - Yes - now resolved - Yes - current - No - I don't know | Indicator variable 7 |
| If “yes,” *Please explain* | |  |
|  | Free text | Indicator variable 7 |
| *Have you ever been told by a doctor or health professional that you have any of the following conditions? Please select all that apply* | | |
|  | - Behavioural or conduct problems - Attention/concentration problems - Anxiety problems - Hearing problems - Vision problems that are corrected by glasses or contact lenses - Vision problems that cannot be corrected by glasses or contact lenses - Brain injury or concussion - Developmental delay - Learning difficulties - Attention deficit hyperactivity disorder (ADHD) - Autism spectrum disorder (ASD) - Epilepsy - Intellectual disability - Mental health problems such as depression, schizophrenia, bipolar disorder, other - Developmental coordination disorder (DCD) - Sensory processing problems - Auditory processing disorder - Alcohol or substance misuse - Memory disorder - Anorexia, bulimia, or other eating disorder | Indicator variable 7 |
| If “mental health problems” selected, *Please specify the mental health problem(s)* | | |
|  | Free text | Indicator variable 7 |
| *Have you ever been told by a doctor or health professional that you have any of the following conditions? Please select all that apply* | | |
|  | - Allergies (to foods, medicines, or the environment e.g. hay fever, peanut allergy) - Asthma - Digestive problems - Arthritis - Skin problems (e.g. eczema) - Eye anomalies or vision problems - Hunger or appetite disturbance - Chronic fatigue syndrome - Diabetes or high blood sugar - Headaches - Migraines - Heart problems - Stroke - Chronic pain - Renal/kidney, bladder or urogenital system problems - Musculo-skeletal problems - Growth problems or short stature - Endocrine and metabolic systems problems - Early onset of puberty - Blood and immune system problems - Difficulty with thermal regulation (controlling own body temperature) - Other [free text] | Indicator variable 7 |
| *Do you have a tremor?* | |  |
|  | - Don’t know - No - Yes | Indicator variable 7 |
| If “yes,” *Please explain* | |  |
|  | Free text | Indicator variable 7 |
| *Do you have a movement disorder?* | |  |
|  | - Don’t know - No - Yes | Indicator variable 7 |
| If “yes,” *What type of movement disorder do you have? Tick all that apply:* | | |
|  | - Abnormal movements or tics - Dystonia - Spastic paraplegia - Ataxia - Developmental coordination disorder (DCD) - Other [free text] | Indicator variable 7 |
