# Supplementary material for: Developmental stuttering with common and complex phenotypes
Source: Dev Med Child Neurol. 2026 Feb 23;68(10):1460–75. doi: 10.1111/dmcn.70208 (PMC13545487; doi:10.1111/dmcn.70208)
Supplement: Supplementary file 14 [file DMCN-68-1460-s014.docx]

**Table S5.**

*Survey questions about development in early life.*

| Developmental domain | Survey response | *n*/total | (%) | |  |
| --- | --- | --- | --- | --- | --- |
| Communication | |  |  | |  |
| First words | Unknown or no response | 155/280 | - | |  |
|  | <15 months | 104/125 | 83.2 | |  |
|  | ≥15 months | 21/125 | 16.8 | |  |
| Short sentences | Unknown or no response | 133/280 | - | |  |
|  | <4 years | 134/147 | 91.2 | |  |
|  | ≥4 years | 13/147 | 8.8 | |  |
| Motor | | | |  | |
| Walking | Unknown or no response | 127/280 | - | |  |
|  | <16 months | 137/153 | 89.5 | |  |
|  | ≥16 months | 16/153 | 10.5 | |  |
| Caregiver concern regarding lost skills | | | |  | |
| Language | Unknown | 83/280 | - | |  |
|  | No concern | 186/197 | 94.4 | |  |
|  | Yes | 11/197 | 5.6 | |  |
| Other skills | Unknown | 61/280 | - | |  |
|  | No concern | 201/217 | 92.6 | |  |
|  | Yes | 16/217 | 7.4 | |  |
| Previous diagnosis | | | |  | |
| Global developmental delay | | 17/326 | 5.2 | |  |
| Intellectual disability | | 10/326 | 3.1 | |  |
| Total | | | |  | |
| No delay known/ reported | | 269/327 | 82.3 | |  |
| Delay in communication or motor milestones | | 33/327 | 10.1 | |  |
| Delay in both communication and motor milestones | | 25/327 | 7.6 | |  |

*Note.* Caregiver concern regarding lost skills indicates *yes* response to “were your caregivers ever concerned that you lost skills for 3 months or more after these skills had been mastered?” Other skills = fine or gross motor, play, social responsiveness.
