# Supplementary material for: Developmental stuttering with common and complex phenotypes
Source: Dev Med Child Neurol. 2026 Feb 23;68(10):1460–75. doi: 10.1111/dmcn.70208 (PMC13545487; doi:10.1111/dmcn.70208)
Supplement: Supplementary file 15 [file DMCN-68-1460-s015.docx]

**Table S2.**

*Demographic variables by participation status for current study.*

|  | Participant (*n*=327) | Non-participant (*n*=1103) | Total invited (*n*=1430) |
| --- | --- | --- | --- |
| Participant type, *n* (%) |  |  |  |
| Adult | 286 (87.5) | 905 (82.1) | 1191 (83.3) |
| Child | 41 (12.5) | 198 (17.9) | 239 (16.7) |
| Age at recruitment to GWAS |  |  |  |
| Mean (SD) in years | 49.1 (21.7) | 42.6 (21.8) | 44.1 (21.9) |
| Median (IQR) in years | 54 (34–67) | 44 (24–62) | 47 (26–64) |
| Range in years | 5–89 | 5–93 | 5–93 |
| Missing, *n* | 0 | 12 | 12 |
| Sex, *n* (%) |  |  |  |
| Male | 231 (70.6) | 740 (67.2) | 971 (67.9) |
| Female | 96 (29.4) | 360 (32.7) | 456 (31.9) |
| Other/undefined | 0 (0.0) | 1 (<0.1) | 1 (<0.1) |
| Missing | 0 (0.0) | 2 (0.2) | 2 (0.1) |
| **Adult participants** |  |  |  |
| Employment status, *n* responded/total (%) | 279/286 (97.6) | 880/905 (97.2) | 1159/1191 (97.3) |
| Full time employment (>30h) | 128 (45.9) | 411 (46.7) | 539 (46.5) |
| Part time employment (<30h) | 30 (10.8) | 104 (11.8) | 134 (11.6) |
| Self-employed (full or part time) | 8 (2.9) | 33 (3.8) | 41 (3.5) |
| Full time student | 20 (7.2) | 85 (9.7) | 105 (9.1) |
| Home/carer duties | 6 (2.2) | 40 (4.5) | 46 (4.0) |
| Volunteer/internship/other unpaid | 12 (4.3) | 19 (2.2) | 31 (2.7) |
| Not currently working | 11 (3.9) | 57 (6.5) | 68 (5.9) |
| Retired | 64 (22.9) | 131 (14.9) | 195 (16.8) |
| Missing | 7 | 25 | 32 |
| Highest qualification, *n* responded/total (%) | 282/286 (98.6) | 892/905 (98.6) | 1174/1191 (98.6) |
| None | 34 (12.1) | 142 (15.9) | 176 (15.0) |
| Trade/vocational training | 19 (6.7) | 90 (10.1) | 109 (9.3) |
| Certificate or diploma | 79 (28.0) | 266 (29.8) | 345 (29.4) |
| Bachelor degree | 69 (24.5) | 215 (24.1) | 284 (24.2) |
| Higher professional | 79 (28.0) | 166 (18.6) | 245 (20.9) |
| Professional accreditation | 1 (0.4) | 11 (1.2) | 12 (1.0) |
| Other | 1 (0.4) | 2 (0.2) | 3 (0.3) |
| Missing | 4 | 13 | 17 |
| Annual salary for full time employment, *n*/total (%) | 119/128 (93.0) | 374/411 (91.0) | 493/539 (91.5) |
| Median (IQR) | $90,000 ($65,000–$120,000) | $80,000 ($60,000–$110,000) | $80,000 ($60,000–$110,000) |
| Range | $21,000–$300,000 | $20,000–$300,000 | $20,000–$300,000 |
| Missing * | 9 | 37 | 46 |
| **Caregivers of child participants** |  |  |  |
| Employment status, *n* responded/total (%) | 21/41 (51.2) | 145/198 (73.2) | 166/239 (69.5) |
| Full time employment (>30h) | 9 (42.9) | 58 (40.0) | 67 (40.4) |
| Part time employment (<30h) | 6 (28.6) | 51 (35.2) | 57 (34.3) |
| Home/carer duties | 3 (14.3) | 18 (12.4) | 21 (12.7) |
| Self-employed (full or part time) | 2 (9.5) | 6 (4.1) | 8 (4.8) |
| Full time student | 1 (4.8) | 8 (5.5) | 9 (5.4) |
| Not currently working | 0 (0.0) | 4 (2.8) | 4 (2.4) |
| Retired | 0 (0.0) | 0 (0.0) | 0 (0.0) |
| Missing | 20 | 53 | 73 |
| Highest qualification, *n* responded/total (%) | 23/41 (56.1%) | 144/198 (72.7%) | 167/239 (69.9%) |
| None | 0 (0.0) | 14 (9.7) | 14 (8.4) |
| Trade/vocational training | 3 (13.0) | 7 (4.9) | 10 (6.0) |
| Certificate or diploma | 8 (34.8) | 42 (29.2) | 50 (29.9) |
| Bachelor degree | 7 (30.4) | 43 (29.9) | 50 (29.9) |
| Higher professional | 5 (21.7) | 38 (26.4) | 43 (25.8) |
| Missing | 18 | 54 | 72 |

*Note*. Demographic data collected when first enrolled into the genome-wide association study and therefore may not reflect demographic status when taking part in the current study. *10/46 missing values for annual salary for full time employment were due to incomplete or ambiguous participant responses.
