# Supplementary material for: Developmental stuttering with common and complex phenotypes
Source: Dev Med Child Neurol. 2026 Feb 23;68(10):1460–75. doi: 10.1111/dmcn.70208 (PMC13545487; doi:10.1111/dmcn.70208)
Supplement: Supplementary file 16 [file DMCN-68-1460-s016.docx]

**Table S1.**

*Indicator variables and response categories used in latent class analysis.*

| Indicator variable & response categories | Data type | Data collection | Rationale |
| --- | --- | --- | --- |
| (1) Family history of stuttering |  |  |  |
| a: No family history  b: Positive family history | Binary | Core survey, pedigree collected during speech assessment | Possible associations between family history of stuttering and clinical outcomes such as age of onset, resolution/ persistence, severity, language abilities (1-7) |
| (2) Age of stuttering onset |  |  |  |
| a: ≤4 years  b: 5–8 years  c: ≥9 years | Ordinal | Core survey | Possible associations between age of onset and clinical outcomes such as resolution/ persistence, severity (1, 8) |
| (3) Stuttering presence and severity |  |  |  |
| a: Resolved stuttering (confirmed during assessment)  b: Mild stuttering (severity rating 1–3 during assessment *or* no stuttering observed during assessment but participant self-reported persistent stuttering)  c: Moderate-to-severe stuttering (severity rating ≥4) | Ordinal | Core survey questions (“have you had a stutter in last 12 months?”), conversation sample during speech assessment | Associations between stuttering presence and severity and clinical outcomes such as negative impact (9, 10) |
| (4) Development in early life |  |  |  |
| a: Typical development  b: Mild or resolved delay (reported delay in either communication *or* motor milestones)  c: Severe or persistent delay (reported delay in both communication and motor milestones, caregiver concern regarding loss of skills, and/or diagnosis of global developmental delay or intellectual disability) | Ordinal | Core survey (previous diagnosis), health and development survey (previous diagnosis, communication and motor milestones) | Possible associations between development in early life and clinical outcomes such as stuttering presence, resolution/ persistence (11-13) |
| (5) Co-occurring speech disorder |  |  |  |
| a: No other speech disorder reported or observed during assessment  b: Mild or resolved speech disorder (reported previous diagnosis of articulation or phonological disorder *or* features of these observed during assessment)  c: Severe or persistent speech disorder (reported previous diagnosis of apraxia of speech or dysarthria *or* features of these observed during assessment) | Ordinal | Core survey (previous diagnosis), health and development survey (previous diagnosis), conversation sample during speech assessment, Oral and Speech Motor Control Protocol during speech assessment | Possible associations between co-occurring speech disorders and clinical outcomes such as resolution/ persistence, severity, response to treatment (14-18) |
| (6) Nonverbal intelligence |  |  |  |
| a: Above average or average scaled score  b: Below average scaled score | Binary | Matrix Reasoning* subtest of Wechsler scales, completed during speech assessment | No associations identified between nonverbal intelligence and clinical outcomes such as resolution/ persistence, severity in children (19-21), but lack of research in adults |
| (7) Co-occurring health conditions |  |  |  |
| a: No diagnoses reported  b: Diagnoses in 1–3 health domains  c: Diagnoses in ≥4 health domains | Ordinal | Core survey (previous diagnosis), health and development survey (previous diagnosis) | Associations between co-occurring health conditions and clinical outcomes such as resolution/ persistence, severity, negative impact, response to treatment (12, 22-25) |

*Note.* Health domains include neurological, immune system, sleep, mental health, neurodevelopmental, cardiovascular system, musculoskeletal system, gut and digestion, endocrine system, and “other” (free text response). *Subtest of Wechsler intelligence scales (WISC-V for children <16 years; WAIS-IV for adults ≥16 years).

**References**

1.	Singer, Hessling A, Kelly EM, Singer L, Jones RM. Clinical Characteristics Associated With Stuttering Persistence: A Meta-Analysis. *Journal of Speech, Language, and Hearing Research*. 2020;63(9):2995.

2.	Walsh B, Christ S, Weber C. Exploring Relationships Among Risk Factors for Persistence in Early Childhood Stuttering. *Journal of Speech Language and Hearing Research.* 2021:1-19.

3.	Darmody T, O’Brian S, Rogers K, Onslow M, Jacobs C, McEwen A, et al. Stuttering, family history and counselling: A contemporary database. *Journal of Fluency Disorders*. 2022;73:105925.

4.	Mohammadi H, Bakhtiary P. Family History of Stuttering among Kurdish Children Who Stutter Near the Age of Onset. *Advanced Biomedical Research*. 2024;13:68.

5.	Buck SM, Lees R, Cook F. The influence of family history of stuttering on the onset of stuttering in young children. *Folia Phoniatrica et Logopaedica*. 2002;54(3):117-24.

6.	Choi D, Conture EG, Tumanova V, Clark CE, Walden TA, Jones RM. Young children’s family history of stuttering and their articulation, language and attentional abilities: An exploratory study. *Journal of Communication Disorders*. 2018;71:22-36.

7.	Boyce JO, Jackson VE, Van Reyk O, Parker R, Vogel AP, Eising E, et al. Self‐reported impact of developmental stuttering across the lifespan. *Developmental Medicine & Child Neurology*. 2022;64(10):1297-306.

8.	Yairi E, Ambrose N. Onset of Stuttering in Preschool Children: Selected Factors. *Journal of Speech and Hearing Research*. 1992;35(4):782-8.

9.	Craig A, Blumgart E, Tran Y. The impact of stuttering on the quality of life in adults who stutter. *Journal of Fluency Disorders*. 2009;34(2):61-71.

10.	Connery A, McCurtin A, Robinson K. The lived experience of stuttering: a synthesis of qualitative studies with implications for rehabilitation. *Disability and rehabilitation*. 2020;42(16):2232-42.

11.	Smith SA, Choo AL, Foster ME. Latent Class Analysis Reveals Distinct Groups Based on Executive Function and Socioemotional Traits, Developmental Conditions, and Stuttering: A Population Study. *Child Psychiatry & Human Development*. 2021:1-17.

12.	Briley PM, Ellis Jr C. The Coexistence of Disabling Conditions in Children Who Stutter: Evidence From the National Health Interview Survey. *Journal of Speech, Language & Hearing Research*. 2018;61(12):2895-905.

13.	Walsh B, Usler E, Bostian A, Mohan R, Lippitt Gerwin K, Brown B, et al. What Are Predictors for Persistence in Childhood Stuttering? *Seminars in Speech & Language*. 2018;39(4):299-312.

14.	Unicomb R, Kefalianos E, Reilly S, Cook F, Morgan A. Prevalence and features of comorbid stuttering and speech sound disorder at age 4 years. *Journal of Communication Disorders*. 2020;84:105976.

15.	Blood GW, Ridenour VJ, Qualls CD, Hammer CS. Co-occurring disorders in children who stutter. Journal of Communication Disorders. 2003;36(6):427-48.

16.	Arndt J, Healey EC. Concomitant Disorders in School-Age Children Who Stutter. *Language, Speech, and Hearing Services in Schools*. 2001;32(2):68-78.

17.	Nippold MA. Phonological and language disorders in children who stutter: impact on treatment recommendations. *Clinical Linguistics & Phonetics*. 2004;18(2):145-59.

18.	Finlay S, Talla PK, Fortin M, Braën J, Levesque L, Verduyckt I. Administering the Lidcombe Program to children who stutter with concomitant disorders: Insights from an exploratory retrospective chart review study. *Journal of Fluency Disorders*. 2025:106101.

19.	Kefalianos E, Onslow M, Packman A, Vogel A, Pezic A, Mensah F, et al. The History of Stuttering by 7 Years of Age: Follow-Up of a Prospective Community Cohort. *Journal of Speech Language and Hearing Research*. 2017;60(10):2828-39.

20.	Yairi E, Ambrose NG, Paden EP, Throneburg RN. Predictive factors of persistence and recovery: Pathways of childhood stuttering. *Journal of Communication Disorders*. 1996;29(1):51-77.

21.	Reilly S, Onslow M, Packman A, Cini E, Conway L, Ukoumunne OC, et al. Natural History of Stuttering to 4 Years of Age: A Prospective Community-Based Study. *Pediatrics.* 2013;132(3):460-7.

22.	Choo AL, Smith SA, Li H. Associations between stuttering, comorbid conditions and executive function in children: a population-based study. *BMC psychology*. 2020;8(1):113.

23.	Unicomb R, Walters J, Choo WL, Hewat S. A retrospective file audit of concomitant disorders reported from a student-led pediatric stuttering clinic. *Speech, Language and Hearing.* 2025;28(1):2416306.

24.	Iverach L, Jones M, O’Brian S, Block S, Lincoln M, Harrison E, et al. The relationship between mental health disorders and treatment outcomes among adults who stutter*. Journal of Fluency Disorders*. 2009;34(1):29-43.

25.	Iverach L, Jones M, McLellan LF, Lyneham HJ, Menzies RG, Onslow M, et al. Prevalence of anxiety disorders among children who stutter. *Journal of Fluency Disorders*. 2016;49:13-28.
