# Supplementary material for: Developmental stuttering with common and complex phenotypes
Source: Dev Med Child Neurol. 2026 Feb 23;68(10):1460–75. doi: 10.1111/dmcn.70208 (PMC13545487; doi:10.1111/dmcn.70208)
Supplement: Supplementary file 17 [file DMCN-68-1460-s017.docx]

**Appendix S1.**

*Core health survey, adult version (genome-wide association study).*

**Introduction to survey:** People who stutter have trouble getting their words out. Stuttering is when people:

- Repeat sounds over and over (e.g. “C-c-c-can I go”)
- Repeat words or syllables over and over (e.g. "Can-can-can-I go")
- Make long prolonged sounds (e.g. "Caaaaaaaaaaaaaan I go")
- Have speech 'stoppages' or 'blocks' where no sounds come out.

| Survey question | Response options | Contribution to statistical analyses |
| --- | --- | --- |
| *At what age did you begin to stutter?* | |  |
|  | - 1-2 years - 3 years - 4 years - 5-6 years - 7-8 years - over 9 years of age | Indicator variable 2 |
| *In the last 12 months, have you had a stutter?* | | Indicator variable 3 |
|  | - No - Yes |  |
| If “no”, *At what age did the stuttering stop or resolve?* | |  |
|  | - 1-2 years - 3 years - 4 years - 5-6 years - 7-8 years - Over 9 years of age | Indicator variable 3 |
| *Have you ever seen a speech and language pathologist for other communication problems, not just stuttering?* | | |
|  | - Don't Know - No - Yes | Indicator variable 5 |
| If “yes,” *Has a speech pathologist ever diagnosed you with any of the following at any time? (tick all that apply)* | | |
|  | - Don’t know/ unsure - Articulation disorder - Apraxia of speech - Dysarthria - Phonological disorder - Expressive language disorder (problems expressing yourself verbally) - Receptive language disorder (problems understanding what is said to you) - Semantic disorder (limited vocabulary) - Grammatical disorder (poor production or understanding of phrases or sentences) - Reading disorder - Spelling disorder - Other [free text] - No diagnosis received | Indicator variable 5 |
| If diagnosis selected, *What was your approximate age when you were first diagnosed with [disorder]?* | | |
|  | Free text | Indicator variable 5 |
| *Apart from stuttering, do you currently experience any problems with communication and/or reading?* | | |
|  | - Don't Know - No - Yes | Indicator variable 5 |
| If “yes,” *Please describe these problems (e.g., difficulty saying certain sounds, mixing up sounds)* | | |
|  | Free text | Indicator variable 5 |
| *Has a doctor or health professional ever told you that you have or have had any of the following? (tick all that apply)* | | |
|  | - Behaviour or conduct problems - Attention/concentration problems - Hearing problems - Vision problems that cannot be corrected by glasses or contact lenses - Brain injury or concussion - Developmental delay - Learning difficulties - Intellectual disability | Indicator variable 7 |
| *Has a doctor or health professional ever diagnosed you with a medical or genetic condition?* | | |
|  | - Don't Know - No - Yes | Indicator variable 7 |
| If “yes,” *Please specify all major medical problem(s) that you have been diagnosed with by a doctor or health professional. Select as many as are appropriate.* | | |
|  | - Arthritis - Asthma - Back problems - Cancer - Chronic fatigue syndrome - Chronic lung disease - Diabetes or high blood sugar - Epilepsy or seizure disorder - Headaches - Heart attack - Heart disease - High blood pressure - HIV infection - Migraines - Neck problems - Rheumatism - Seasonal allergies - Stroke - Tuberculosis - Stomach ulcers - (Any other) chronic pain - Other [free text] | Indicator variable 7 |
| *Has a doctor or health professional ever diagnosed you with any mental health or behaviour problem?* | | |
|  | - Don't Know - No - Yes | Indicator variable 7 |
| If “yes,” *Please specify all major mental health or behaviour problem(s) that you have been diagnosed with by a doctor or health professional. Select as many as are appropriate.* | | |
|  | - Alcohol or other substance misuse - Anorexia - Anxiety - Attention deficit hyperactivity disorder (ADHD) - Autism spectrum disorder (ASD), Asperger’s - Bipolar disorder - Bulimia - Conduct disorder or Oppositional disorder - Depression - Drug addiction - Another eating disorder (i.e., not anorexia or bulimia) - Manic depression - Memory disorder - Narcolepsy - Obsessive compulsive disorder - Premenstrual dysphoric disorder (PMDD) - Psychosis - Post-traumatic stress disorder (PTSD) | Indicator variable 7 |
| If diagnosis selected, *How old were you when you were first told that you had [diagnosis]?* | | |
|  | Free text | Indicator variable 7 |
| *Does anyone in your family stutter?* | |  |
|  | - Don't Know - No - Yes | Indicator variable 1 |
| If “yes,” *Please specify who and how they are related to you* | |  |
|  | - Mother - Father - Sister - Brother - Daughter - Son - Maternal aunt - Maternal uncle - Female cousin (maternal) - Male cousin (maternal) - Maternal grandmother - Maternal grandfather - Paternal aunt - Paternal uncle - Female cousin (paternal) - Male cousin (paternal) - Paternal grandmother - Paternal grandfather - Other [free text] | N/A |
