# Supplementary material for: Developmental stuttering with common and complex phenotypes
Source: Dev Med Child Neurol. 2026 Feb 23;68(10):1460–75. doi: 10.1111/dmcn.70208 (PMC13545487; doi:10.1111/dmcn.70208)
Supplement: Supplementary file 18 [file DMCN-68-1460-s004.docx]

**Figure S1.**

*Participant recruitment and selection of final analytic sample.*
